# *B3GALT6* mutations lead to compromised connective tissue biomechanics in Ehlers-Danlos syndrome

**DOI:** 10.1172/jci.insight.179474

**Published:** 2025-08-22

**Authors:** Roméo Milan Diana, Benjamin Jolivet, Jean-Baptiste Vincourt, Sébastien Hergalant, Grégory Francius, Yasaman Karami, Hamed Khakzad, Rebekka Wild, Marie Bourgeais, Anne Robert, Alison Wurtz, Guillermo Barreto, Nick Ramalanjaona, Déborah Helle, Rachel Onifarasoaniaina, Sophie Front, Chrystel Lopin-Bon, Delfien Syx, Fransiska Malfait, Sylvie Fournel-Gigleux, Sandrine Gulberti, Catherine Bui

**Affiliations:** 1 Université de Lorraine, CNRS, IMoPA, UMR 7365, F-54000 Nancy, France.; 2 Université de Lorraine, Inserm, NGERE, U1256, F-54000 Nancy, France.; 3 Université de Lorraine, CNRS, LCPME, UMR 7564, F-54000 Nancy, France.; 4 Université de Lorraine, CNRS, Inria, LORIA, F-54000 Nancy, France.; 5 Institut de Biologie Structurale, UMR 5075, Université Grenoble Alpes, CNRS, CEA, 38000 Grenoble, France.; 6 Institut Cochin, INSERM U1016, CNRS UMR 8104, Université de Paris, Paris, France.; 7 Institut de Chimie Organique et Analytique, UMR 7311, Université d’Orléans et CNRS, Rue de Chartres, BP 6759, 45067 Orléans Cedex 2, France.; 8 Center for Medical Genetics, Ghent University Hospital, and Department of Biomolecular Medicine, Ghent University, Belgium.

**Keywords:** Cell biology, Genetics, Extracellular matrix, Genetic diseases, Glycobiology

## Abstract

Ehlers-Danlos syndromes (EDS) comprise a genetically and clinically heterogenous group of rare diseases that cause severe, often fatal, damage to connective tissue. The molecular basis of EDS implicates defects in extracellular matrix components, including various fibrillar collagens and glycosaminoglycans (GAGs). However, the precise pathogenic mechanisms behind EDS remain elusive. Here, we have implemented a multi-tiered approach to demonstrate the functional impact of *B3GALT6* mutations on biochemical and developmental processes, ultimately leading to the spondylodysplastic subtype of EDS (spEDS), characterized by severe musculoskeletal symptoms. We show that the loss of function of β1,3-galactosyltransferase 6 (β3GalT6) is partially compensated by β1,3-glucuronosyltransferase 3 (GlcAT-I), the next enzyme in the GAG biosynthetic pathway. In addition, results from transcriptomics, collagen analysis, and biophysical experiments revealed that impaired collagen maturation, including defective glycosylation of collagen XII, contributes to altered tissue structure and biomechanics, the hallmarks of spEDS. Our findings unravel a new pathogenic mechanism of spEDS and bring us one step closer to therapeutic strategies, including cell and tissue engineering.

## Abstract 2

*B3GALT6* pathogenic variants impair the biomechanics of connective tissue, despite a compensation mechanism of glycosaminoglycan synthesis

## Introduction

The Ehlers-Danlos syndromes (EDS) comprise a clinically and genetically heterogenous group of heritable connective tissue disorders characterized by joint hypermobility, skin hyperextensibility, and organ/vascular fragility. Besides defects in fibrillar collagens and their modifying enzymes, the molecular heterogeneity of EDS has been expanded to enzymes involved in glycosaminoglycan (GAG) biosynthesis and other extracellular matrix (ECM) components (i.e., tenascin-X and collagen XII (Col XII)) (1, 2). Because the collagen architecture and the ECM functional properties are affected in EDS, it is considered a “collagen-related disorder” (2). Defects in GAG biosynthesis are severe, from strongly disabling to lethal (3) and overlap with other osteochondrodysplasias, cutis laxa, hereditary myopathies, and TGF-β–related disorders (4). Pathogenic variants of *B3GALT6* (NM_080605.3), coding for β1,3-galactosyltransferase 6 (β3GalT6, B3GT3, EC 2.4.1.134), compromise the early steps of GAG synthesis. These variants are classified in the spondylodysplastic EDS (spEDS) subtype (1), in which *B4GALT7* and *SLC39A13* pathogenic variants are also included (1–3). Due to their low incidence, spEDS remain poorly explored, leading to delayed, sometimes erroneous diagnosis and late patient management.

GAGs are long linear sulfated polysaccharides attached to various core proteins forming proteoglycans (PGs), which exert versatile functions in multiple pathophysiological conditions (5, 6). They play essential roles in embryonic and postnatal development of connective tissues (CTs) such as skin, bone, cartilage, muscle, blood vessels, and organs (7). GAGs are classified according to the composition of sugar repeats and include heparan-sulfates (HS) and chondroitin/dermatan-sulfates (CS/DS). PGs are ubiquitously expressed at the plasma membrane and in the ECM of all animal cells and tissues and exert critical functions in cell-cell and cell-matrix communication, cell signaling, and tissue homeostasis. They act as a reservoir for multiple growth factors and mediators (8). GAGs also play important roles in the ECM assembly and function by interacting with other matrix components, such as collagens, thereby ensuring the peculiar architecture and biomechanical properties of CTs (5).

The biosynthesis of GAG chains requires the concerted action of multiple enzymes. These include glycosyltransferases (GTs), which initiate and elongate the saccharide backbone, and sulfotransferases, epimerases, kinases, and phosphatases, which complete their maturation (9). This machinery is conserved over a wide range of eukaryotic organisms and its alteration is associated with multiple pathological conditions, including cancers (6, 10, 11), immune diseases (12), and genetic diseases (2). The assembly of HS and CS/DS chains is initiated by the synthesis of a tetrasaccharide linker, Glucuronic acid-β1,3-Galactose-β1,3-Galactose-β1,4-Xylose-β1 (GlcA-β1,3-Gal-β1,3-Gal-β1,4-Xyl-β1) attached to specific serine residues of the core protein, which is necessary for the elongation of the GAG chains. β3GalT6 is a Golgi membrane-bound GT that catalyses the transfer of Gal from UDP-Gal to the Gal-β1,4-Xyl disaccharide linked to the core protein of a PG (Supplemental Figure 1; supplemental material available online with this article; https://doi.org/10.1172/jci.insight.179474DS1). Therefore, it is a key player in the initiation of GAG chains (13).

Pathogenic variants of genes encoding the enzymes implicated in the formation of the tetrasaccharide linker, including β3GalT6, lead to a spectrum of severe conditions called linkeropathies (Supplemental Figure 1). In a pioneering study, we identified *B3GALT6* pathogenic variants in 5 individuals from 3 Iranian families carrying a homozygous c.619G>C p.(D207H) mutation (patients P1 and P2), a homozygous c.649G>A p.(G217S) mutation (patient P5) or a compound heterozygous c.619G>C p.(D207H)/c.323_344del p.(Ala108Glyfs*163) frameshift mutation (patients P3 and P4) (14). These defects were associated with extensive skeletal abnormalities, bone fragility, mild to moderate intellectual deficit, and impaired wound healing classified as spEDS (14). Simultaneously, Nakajima et al. (15) identified mutations cosegregating with spondyloepimetaphyseal dysplasia with joint laxity type 1 (SEMD-JL1) and spEDS in 2 small cohorts of Eastern Asian families. These findings underscore the pleiotropic consequences of defective β3GalT6 on processes that control the development and physiology of skin, bone, tendon, and ligaments. In a following study, we expanded the spEDS clinical spectrum to cardiac defects, cervical spine instability, respiratory insufficiency, and cerebrovascular accidents (16). Today, the list of *B3GALT6* variants has grown to 48 mutations affecting 70 individuals (54 families), representing a large sample of affected individuals (2, 17–21). These conditions are inherited in an autosomal recessive manner except for 1 dominant variant that cosegregated with moderate malformations (20). Together, the conditions caused by *B3GALT6* mutations comprise multisystemic disorders, including spEDS-*B3GALT6* (formerly type 2 spEDS, EDSSPD2), SEMD-JL1, and Al-Gazali syndrome (1, 15, 22, 23).

To date, our understanding of how *B3GALT6* mutations affect protein function and GAG synthesis and how these defects cause clinical outcomes, is limited. Herein, we selected 3 pathogenic variants p.(Y182C), p.(D207H) and p.(G217S) and established their impact at multiple levels, from gene defect to phenotype (Supplemental Table 1). We demonstrate that all 3 mutations provoke a complete loss of function (LOF) of recombinant β3GalT6 expressed in *Escherichia coli* (*E*. *coli*). Using molecular dynamics (MD) simulations, we identified a common structural alteration of the catalytic site caused by these mutations. Surprisingly, despite β3GalT6 LOF, patient fibroblasts and 2 CRISPR-Cas9–KO cell models retained the capacity of synthesizing GAG chains, although their content and profile were severely compromised. This residual GAG synthesis could in part be attributed to the (β1,3-glucuronosyltransferase 3, GlcAT-I, EC 2.4.1.135), which is involved in the formation of the tetrasaccharide linker. Furthermore, transcriptomic analyses revealed that the most dysregulated pathways were those involved in collagen maturation. A cell model of ECM synthesis revealed altered structure and biomechanics upon *B3galt6* extinction, reminiscent of the clinical situation, plausibly because Col XII, a Fibril Associated Collagen with Interrupted Triple helices (FACIT) also known as a PG, was devoid of GAG substitution. Our findings indicate that *B3GALT6* mutations severely compromise tissue biomechanics, despite residual GAG synthesis. Additionally, we have discovered a previously unknown connection between GAG synthesis and collagen maturation, which may have a significant impact on the clinical expression of spEDS and advance our understanding of its pathobiology.

## Results

### Disease-associated missense mutations impair β3GalT6 activity due to common structural defects.

**Figure 1:**
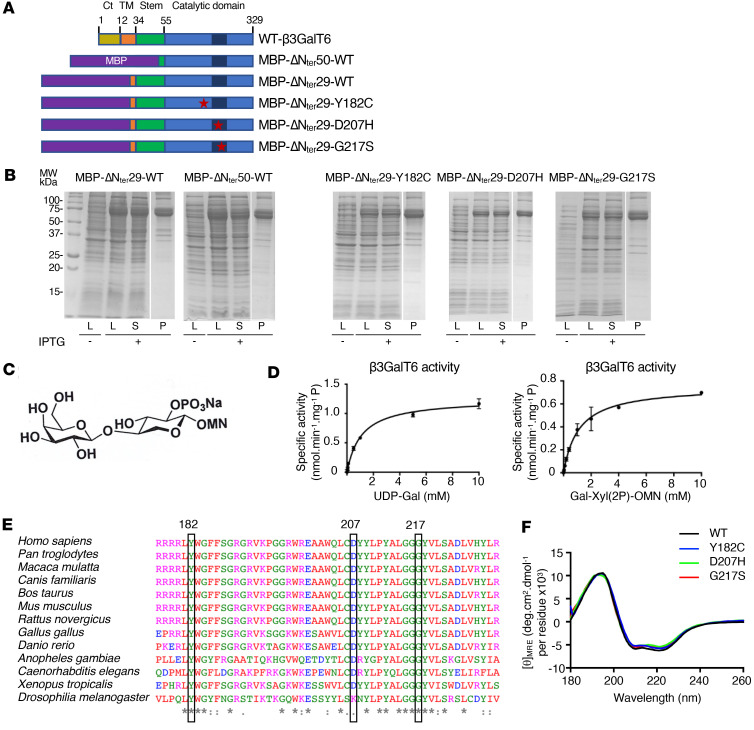
Mutant β3GalT6 proteins were purified at similar yields as the WT and display a loss of enzymatic activity without large structural protein alterations. (**A**) Schematic representation of the WT and mutant b3GalT6 proteins. The maltose-binding protein (MBP) is in purple. (**B**) Expression of WT and mutant MBP-ΔN_ter_29-b3GalT proteins in *E*. *coli*. From left to right, cell lysates (L) before and after induction with IPTG, supernatant (S), and purified protein fraction (P). (**C**) Chemical structure of the synthetic phosphorylated acceptor substrate Gal-Xyl(2P)-OMN used in GT assays. (**D**) Michaelis-Menten kinetics of MBP-b3GalT6ΔN_ter_29-WT toward the donor substrate UDP-Gal and the acceptor substrate Gal-Xyl(2P)-OMN. Data are expressed as mean ± SD (*n* = 3 protein batches in duplicate). (**E**) Multiple sequence alignment of the catalytic domain of WT β3GalT6 from different species. Protein sequences are from *Homo sapiens* (UniProtKB:Q96L58), *Pan troglodytes* (UniProtKB:H2PXT4), *Macaca mulatta* (UniProtKB:F7CX70), *Canis familiaris* (UniProtKB:Q257A0), *Bos taurus* (UniProtKB:F1MVH6), *Mus musculus* (UniProtKB:Q91Z92), *Rattus norvegicus* (UniProtKB:D3ZQC1), *Gallus gallus* (UniProtKB:A0A8V0ZK44), *Danio rerio* (UniProtKB:Q256Z7), *Anopheles gambiae* (NCBI:XP_319940.4), *Caenorhabditis elegans* (UniProtKB:Q9N491), *Xenopus tropicalis* (UniProtKB:Q5BL85), *Drosophilia melanogaster* (UniProtKB:A1Z7G9). Residues are coloured according to their physicochemical properties by the Clustal Omega software (68). The mutated residues are framed. (**F**) Far-UV CD spectra of MBP-b3GalT6ΔN_ter_29-WT (black), MBP-b3GalT6ΔN_ter_29-Y182C (blue), MBP-b3GalT6ΔN_ter_29-D207H (green) and MBP-b3GalT6ΔN_ter_29-G217S (red). Data are mean of 3 measurements per protein.

To determine the consequences of the mutations p.(Y182C), p.(D207H), and p.(G217S) encountered in spEDS-*B3GALT6* patients (P2, P3 and P5, [14]) or SEMD-JL1 (PVII.1) (16) (Supplemental Table 1), we expressed and purified the recombinant WT and mutant β3GalT6 proteins in *E*. *coli*. We analyzed their activity and determined, when possible, their kinetic parameters. β3GalT6 is a type II transmembrane protein with a cytoplasmic (Ct) N-terminus (N_ter_) domain, a single pass transmembrane (TM) helix, an unstructured stem region, and a globular catalytic luminal domain. Two constructs lacking both the Ct domain and the TM helix (MBP-DN_ter_29-WT), or in addition, the stem region (MBP-DN_ter_50-WT), were generated (Figure 1A) and led to the expression of soluble proteins (Figure 1B). Both truncated proteins were active towards the disaccharide Gal-β1,4-Xyl(2-*O*-phosphate)-*O*-7-methoxy-naphthyle (Gal-Xyl(2P)-OMN) (Figure 1C), but MBP-DN_ter_29-WT activity was higher (Figure 1D and Supplemental Figure 2). *K*_m_ value towards Gal-Xyl-(2P)-OMN was similar to those previously reported (24) (Table 1). Therefore, mutant proteins were generated from this construct and produced to similar yields as the WT (Figure 1B). Strikingly, all 3 pathogenic variants, p.(G217S) (homozygous in patient P5), p.(D207H) (homozygous in patient P2 and heterozygous with a frameshift mutation in patient P3), and p.(Y182C) (homozygous in patient PVII.1) caused a complete LOF of the recombinant enzymes (Table 1). This result is consistent with the conserved status of these 3 residues across species (Figure 1E), emphasizing their key functional and/or structural role. To assess whether they may induce large structural protein alterations, circular dichroism (CD) experiments were performed. The far-UV spectra of β3GalT6 mutants were similar to the WT and display a classic α-helix signature with a maximum at approximately 190 nm and 2 minima at approximately 210 nm and approximately 220 nm (Figure 1F). This suggests that the overall conformation of each mutant is comparable to the WT, ruling out the hypothesis of large structural changes causing the LOF.

**Figure 2:**
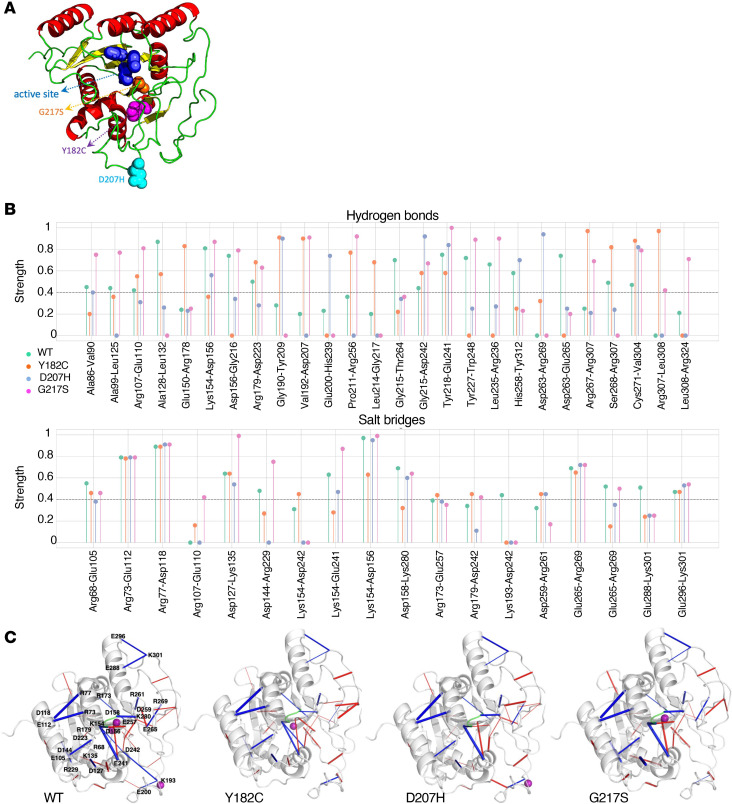
Structure prediction of the interaction network of WT and mutant β3GalT6. (**A**) AlphaFold model of β3GalT6. The ^156^DDD^158^ motif is shown as blue spheres; mutated residues Y182C, D207H, and G217S are in purple, turquoise, and orange, respectively. (**B**) H-bond (top) and salt bridge (bottom) strengths, shown as the percentage of conformations in which each interaction occurs for WT and mutants. (**C**) Interaction networks of WT and mutants. Key interacting residues are shown as magenta spheres and the ^156^DDD^158^ motif as a green line. Red and blue lines indicate H-bonds and salt bridges, respectively, with line thickness proportional to interaction frequency. Interacting residues are labeled on the WT model.

Since the structure of β3GalT6 is unresolved, molecular modeling was used to localize disease-associated mutations. AlphaFold predictions suggest an α/β/α-like organization characteristic of GTs with a GT-A fold (Figure 2A) (24), with 9 α-helices and 2 β-sheets: one with strands β1–β4 and β6–β8, and a second with β5 and β9 (Supplemental Figure 3, A and B). The positions of the disease-associated mutations (Y182C, D207H, and G217S) are shown in the 3D model, as well as the sequence alignment with β1,3-*N*-acetylglucosaminyltransferase 2 (B3GNT2) (Figure 2A and Supplemental Figure 3C). The G217 residue lies in the strand β7 buried in the active site, near the D×D motif (^156^DDD^158^), critical for Mn²^+^ coordination and UDP-Gal binding (25). Y182 is within β6, also buried in the active site, while D207 is located in a loop (G188-P211) at the periphery of the catalytic domain (Figure 2A).

To assess the impact of these mutations on β3GalT6 dynamics, for each of the WT and three mutants (Y182C, D207H and G217S) we performed three replicates of 1 μs MD simulations, leading to a total of 12 μs simulation time. Analysis of hydrogen bonds (H-bonds) and salt bridges revealed interaction networks within the catalytic domain. Figure 2B (upper panel) shows H-bonds with greater than or equal to 40% strength variation in at least one system; salt bridges occurring in more than 40% of simulation time are shown in the lower panel. These interactions were mapped onto the protein structures (Figure 2C).

Comparison of WT and mutants revealed the consistent loss of a K193–D242 salt bridge in all 3 variants (Figure 2C). This salt bridge links the G188–P211 loop (between β6 (L181-S187) and β7 (Y212-S221)) and helix α8 (E241–L249), likely contributing to local structural stability and ligand interactions. Its loss may underlie the LOF phenotype induced by the pathogenic variants. Supporting this, residual fluctuations analysis (Supplemental Figure 4) showed the R191–D207 segment within the G188–P211 loop exhibited high fluctuations, with reduced amplitudes in the mutants relative to WT. These changes of dynamic behavior could critically alter β3GalT6 function.

Some interactions were mutation specific. Y182C and G217S showed slightly fewer interactions than WT, while D207H showed the least (Figure 2, B and C). Regarding the ^156^DDD^158^ motif, WT featured 2 salt bridges (K154–D156 and D158–K280) and 1 H-bond (D156–G216), which were retained in G217S and D207H but reduced or absent in Y182C. Overall, disruption of the K193–D242 critical salt bridge that stabilizes the G188-P211 flexible long loop and mutation-specific changes in interaction strength likely contribute to the LOF observed in the 3 variants. Further structural studies, including crystallization of WT β3GalT6, are needed to confirm these findings.

### β3GalT6 LOF in patient fibroblasts and in B3GALT6-KO cells is partially compensated and depends on the cellular context.

**Figure 3:**
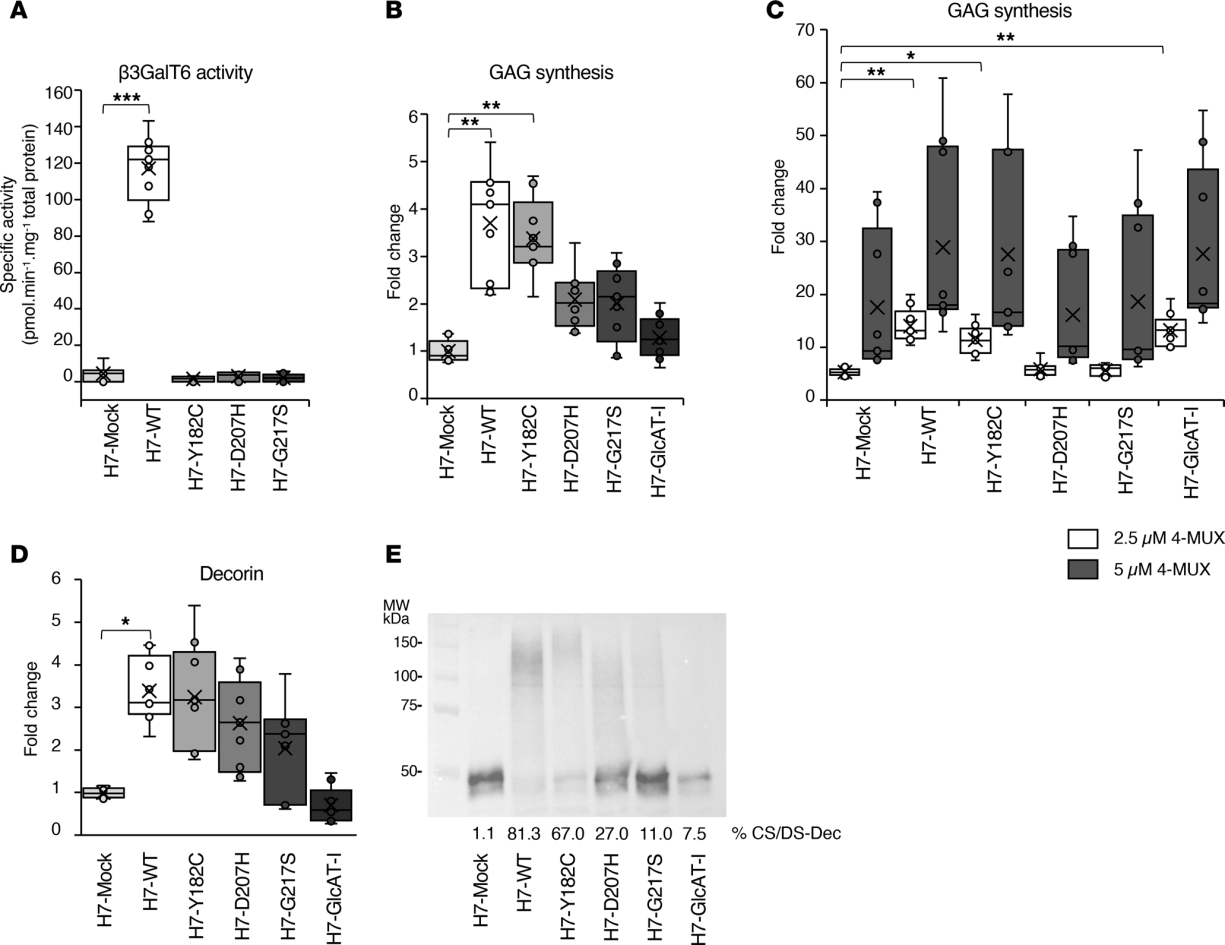
GAG synthesis is strongly reduced but not abolished in *B3GALT6-KO* H7. (**A**) In vitro β3GalT6 activities using total cell lysates. Data are presented as a box plot with the mean symbolized by an X (*n* = 3 independent experiments in triplicate). (**B**) Level of radiolabeled endogenous GAG chains in *B3GALT6*-KO cells expressing WT, mutant β3GalT6, and GlcAT-I. (**C**) Level of radiolabeled 4-MUX-primed GAG chains in *B3GALT6*-KO cells expressing WT, mutant β3GalT6, and GlcAT-I. (**D**) Level of radiolabeled decorin in cotransfected *B3GALT6*-KO cells. For **B**–**D**, data are presented as a box plot with the mean fold-change symbolized by a cross (*n* = 3 independent experiments in triplicate). All fold-changes were calculated by dividing the radioactivity level (dpm/ng of DNA) for each GT to the value obtained for H7-Mock cells. (**E**) Representative immunoblot of decorin in cotransfected *B3GALT6*-KO cells. CS/DS-Dec corresponds to the GAG-substituted decorin (*n* = 3 independent experiments); *P < 0.05; **P < 0.01; ***P < 0.001 (1-way ANOVA).

To investigate the striking effect of these mutations on the activity of purified β3GalT6, we used the *B3GALT6*-KO H7 clone as a KO model, for which the galactosyltransferase activity was very low and protein expression undetectable (Supplemental Figure 5B and Supplemental Figure 6A). After reexpression of the different enzymes in this model, we measured the activities toward Gal-Xyl(2P)-OMN using the cell lysates. Our results indicate that enzyme activity could only be detected in *B3GALT6*-KO cells expressing the WT form, whereas the mutant-expressing and H7-mock cells had similar enzymatic activities, just above the detection level (Figure 3A). Similarly, the enzyme activity in patient fibroblasts (P2, P3, and P5) was dramatically reduced and considered as residual activity (Supplemental Figure 7). We also confirmed that the protein expression of WT and β3GalT6 mutants was similar and that proteins were colocalized with the cis-Golgi marker GM130 (Supplemental Figure 6). Taken together, our data confirm that the 3 mutations cause a complete LOF of β3GalT6 in purified proteins and barely detectable enzyme activities in patient fibroblasts and mutant-expressing *B3GALT6*-KO cells.

Previous analyses of CS/DS and HS in patient fibroblasts by us and others suggested that GAG synthesis was strongly impaired but still present (14–16) (Supplemental Table 1). To investigate this discrepancy, we analyzed the capacity of the *B3GALT6*-KO cells to synthesize GAGs after the reexpression of WT or β3GalT6 mutants. Figure 3B shows that the endogenous GAG synthesis was significantly increased in the H7-WT (4-fold), in the H7-Y182C (3-fold) cells compared with the H7-mock cells, indicating either a residual enzyme activity that couldn’t be detected in vitro previously and/or the existence of a compensation mechanism in cellulo. On the other hand, an endogenous and residual GAG synthesis was also observed in the cells expressing H7-D207H, H7-G217S, and GlcAT-I, but no significant increase was measured. Then, the exogenous xyloside 4-methylumbelliferyl-β-D-xylopyranoside (4-MUX) was used to initiate GAG synthesis via β4GalT7, the enzyme preceding β3GalT6 in the GAG synthesis. The results showed that the synthesis of the GAG chains primed by 4-MUX (2.5 μM) was significantly increased for the WT, the H7-182C, and the H7-GlcAT-I cells, which previously had the lowest endogenous GAG synthesis (Figure 3C). Our results demonstrate that the synthesis of GAG chains is increased in a dose-dependent manner in transfected *B3GALT6*-KO, including the H7-mock cells. However, the differences in GAG synthesis were no longer significant at the highest dose of 4-MUX.

To further analyze how *B3GALT6* inactivation or mutations affect in cellulo CS/DS synthesis, we examined the level of radiolabeled decorin (Figure 3D) and its glycosylation by immunoblot (Figure 3E). In Figure 3D, the fold-changes in the level of radiolabeled decorin were consistent with the immunoblot analysis, where higher fold-changes were associated with GAG-substituted decorin. Immunoblot analysis of the decorin in H7-WT cells exhibited a major broad band (> 100 kDa), corresponding to the GAG-substituted PG and a faint signal (~ 50 kDa) corresponding to the unsubstituted core protein (Figure 3E). Consistent with the endogenous GAG synthesis and the level of radiolabeled decorin, the H7-Y182C cells exhibited a major band corresponding to the GAG-substituted decorin, which also appears to have longer GAG chains. In the H7-mock cells, the unmodified core-protein was the major band as well as in the H7-D207H and H7-G217S, although notably, the presence of a broad band of lower intensity was clearly seen for these mutants (Figure 3E). Consistent with Figure 3, B–D, small quantity of GAGs was detected in H7-GlcAT-I cells. However, GAG biosynthesis was restored in the presence of an excess of 4-MUX, suggesting that the compensation mechanism occurring in H7-GlcAT-I cells is likely dependent on the nature and availability of the acceptor substrate.

The radiolabeled GAG chains of decorin were also analyzed in electrophoretic gels (Supplemental Figure 8) and show that the H7-mock cells and those transiently expressing the different GT, are still able to initiate GAG synthesis onto the core protein of decorin to various degrees in cellulo despite the LOF observed in vitro. This suggests that the residual GAG synthesis is maintained via an alternative pathway and highlights the fine-tuning of this process, depending on the level of GTs expression and the availability and the nature of the acceptor substrate.

**Figure 4:**
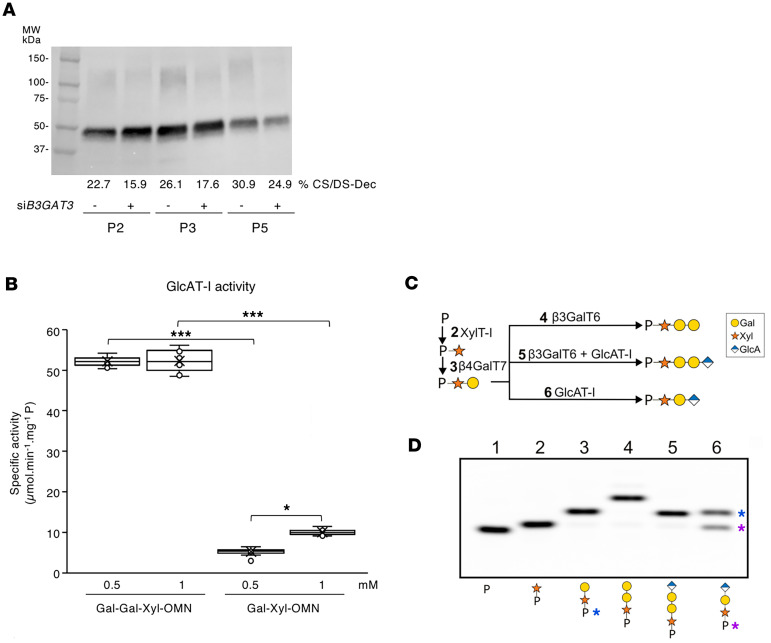
GlcAT-I is implicated in the synthesis of the noncanonical trisaccharide linkage region and contributes to residual GAG synthesis. (**A**) Representative immunoblot of endogenous decorin in spEDS patient fibroblasts following *B3GAT3* silencing. CS/DS-Dec corresponds to the GAG-substituted decorin (*n* = 3 independent experiments). (**B**) In vitro activity of the recombinant purified GlcAT-I. Data are presented as box plot with the mean symbolized by an X (*n* = 3 assays, one triplicate per concentration) *P < 0.05; ***P < 0.001 (1-way ANOVA). (**C**) Diagram of enzymatic reaction steps to produce the native tetrasaccharide linker [GlcAβ1–3Galβ1–3Galβ1–4Xyl] (reaction 5) or the noncanonical trisaccharide [GlcA-3Galβ1-4Xyl] linker (reaction 6). Monosaccharide symbols follow the symbol nomenclature for Glycans system (69) (**D**) Stepwise glycan addition onto the peptide (P). Lane 1 contains the unmodified peptide (P) while lanes 2–6 contain reaction products as outlined in **C**. Lane 6 contains the [Galβ1–4Xyl]-peptide (blue asterisks) and the [GlcA-3Galβ1-4Xyl]-peptide products (purple asterisks).

A plausible hypothesis is the formation of a noncanonical trisaccharide linker (GlcA-β1,3-Gal-β1,4-Xyl-β1) as an alternate primer for GAG synthesis, as reported in *B3GALT6*-deficient patient urine (26) and in a KO-zebrafish model (27). To confirm this, patient fibroblasts P2, P3 and P5 were transfected by a silencing RNA (siRNA) targeting *B3GAT3* mRNA. Results show that, upon knockdown, the GAG-substitution of decorin was further reduced (Figure 4A). We next expressed and purified a truncated GlcAT-I and evaluated its activity towards the Gal-β1,3-Gal-β1,4-Xyl-β-*O*-7-methoxynaphthyle (Gal-Gal-Xyl-OMN) and Gal-β1,4-Xyl-β-*O*-7-methoxynaphthyle (Gal-Xyl-OMN) analogue substrates. Results in Figure 4B indicate that GlcAT-I was highly active towards the trisaccharide Gal-Gal-Xyl-OMN, which mimics the canonical linker. In addition, the enzyme exhibited a lower (about 5-times) but significant activity towards the disaccharide Gal-Xyl-OMN (1mM). Next, in vitro glycosylation assays were performed as shown in Figure 4C. The step-wise addition of the first 3 glycans, leading to the formation of a [Galβ1–3Galβ1–4Xyl]-peptide, resulted in slower migration due to the increased molecular mass (Figure 4D). In presence of GlcAT-I, the full native linker, [GlcAβ1–3Galβ1–3Galβ1–4Xyl] was produced, as observed by an increase in migration speed due to the addition of a negative charge. Interestingly, for reaction mix 6 (which was without β3GalT6), the partial conversion of the [Galβ1–4Xyl]-peptide by GlcAT-I was observed as an additional band on the gel. The identities of the reaction products were confirmed by MALDI-TOF analysis (Supplemental Figure 9). Taken together, our data demonstrate that GlcAT-I can produce a noncanonical trisaccharide linker from different acceptor substrates when β3GalT6 activity is defective, and it contributes to residual GAG synthesis in a pathological context.

### Transcriptomics reveals dysregulated collagen maturation in fibroblasts from patients with spEDS.

**Figure 5:**
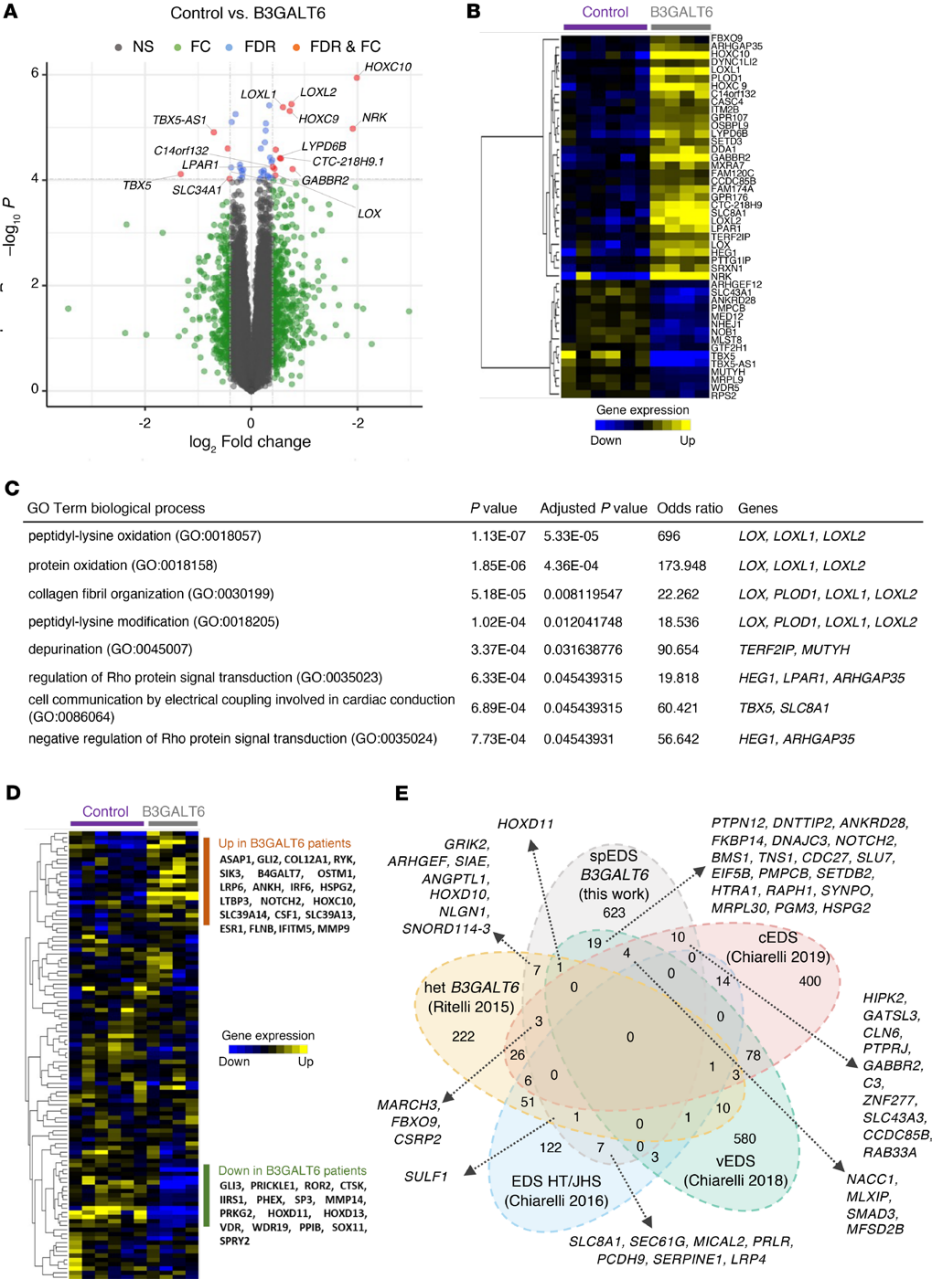
Transcriptome analysis of dermal fibroblasts from patients with spEDS reveals dysregulated collagen maturation and developmental pathways. (**A**) Volcano-plot displaying the most differentially expressed genes (DEG) between spEDS (*B3GALT6*) patient and control individual fibroblasts. Each dot represents a transcript probe set. *X*-axis, differential expressions (fold-changes); *Y*-axis, differential statistics (moderated t-tests). FC, fold-change; FDR, false discovery rate. (**B**) Hierarchical clustering heat map showing a signature of 52 significantly dysregulated transcripts between patients with spEDS (*B3GALT6, n* = 4 samples from 3 spEDS patients, including one replicate) and control individuals (*n* = 6 samples). Yellow, upregulated genes; blue, downregulated genes; black, median expression level. (**C**) Top functional annotations and affected pathways in patients with spEDS. (**D**) Hierarchical clustering heat map showing relative expression levels of 107 mRNAs representing the spondylodysplastic signature (as accessed from the OpenTargets database in July 2022). Yellow, upregulated genes; blue, downregulated genes; black, median expression level. (**E**) Venn diagram depicting the amount of DEG overlap and uniqueness between EDS transcriptome microarray experiments (human Affymetrix platform) performed from dermal fibroblasts. DEG list cutoffs: *P* < 0.005, with a maximum of 700 genes. See Methods for information on each transcriptome.

We next examined the global impact of *B3GALT6* pathogenic variants on biological pathways. We performed a gene expression profiling of fibroblasts from patients P2 and P5 (homozygous for p.(D207H) and p(G217S), respectively), and P3 (harboring p.(D207H) and p.(A108Gfs*163) mutations) (Supplemental Table 1) versus 6 individuals who acted as controls. Comparison of transcriptomes from controls and patients with spEDS revealed 2 groups of dysregulated genes (differentially expressed genes (DEG), false discovery rate (FDR) < 0.1) with 11 genes reaching transcriptome-wide statistical significance (FDR < 0.05, in red dots, Figure 5A and Supplemental Table 3). Among them, *LOXL1*, *LOXL2*, *LOX*, and *PLOD1*, coding for Lysyl Oxidase Homolog 1, Lysyl Oxidase Homolog 2, Lysyl Oxidase, and procollagen-lysine 2-oxoglutarate 5-dioxygenase 1 (LH1), respectively, all involved in collagen maturation, exhibited higher expression in spEDS samples compared with controls. Using targeted quantitative PCR (qPCR), we showed a decreased expression of *LOXL1* mRNA in fibroblasts from patient P2 while an increased expression of both *LOXL1* and *LOXL2* mRNA in fibroblasts from patients P3 and P5 (Supplemental Figure 10).

In addition, *HOXC9*, *HOXC10*, as well as *TBX5*, all coding for transcription factors playing wide roles in morphogenesis during development, were among the most up- and downregulated genes in spEDS fibroblasts. Further hierarchical clustering of the top DEG (FDR < 0.1) clearly delineated 2 clusters of strongly correlated gene expression profiles, with a majority of upregulated genes in patients who had *B3GALT6*-spEDS (Figure 5B). This set of dysregulated genes, including those highlighted in the volcano plot (Figure 5A), i.e., *LOXL1*, *LOXL2*, *LOX*, *HOXC9*, *HOXC10*, and *TBX5*, represents a specific transcriptomic signature for these patients with spEDS. Subsequent gene ontology (GO) and pathway analyses of DEG pointed out enriched biological processes relevant to collagen maturation and organization, such as peptidyl-lysine oxidation, collagen fibril organization, and peptidyl-lysine modification (Figure 5C and Supplemental Table 4). The GO analyses revealed a disturbance in the Rho signaling pathway, consistent with the role of Rho GTPases as key integrators of ECM signaling (28). This observation is relevant to the disturbed ECM assembly in patients with spEDS.

We also evaluated our results against a spEDS gene signature compiled from the literature and comprising 107 genes known to be associated with spEDS (from the OpenTargets platform, under EDS, spondylodysplastic type, score>0.02; https://platform.opentargets.org/). Hierarchical clustering performed with this gene set allowed the extraction of 2 different gene clusters, corresponding to a quarter of the public spEDS signature. Therefore, a specific spEDS profile (Figure 5D) could be refined from our study, allowing the identification of genes relevant in the pathogenesis of spEDS. From this analysis, *COL12A1* was identified in the upregulated gene cluster, emphasizing the potential role of Col XII in matrix alteration.

We next compared our data to 4 other microarray transcriptomes from previous studies, including (a) 2 *B3GALT6*-deficient siblings (29), GSE58312), (b) a cohort of patients with joint hypermobility syndrome/EDS hypermobility type (JHS/EDS-HT) (30), PMID: 27518164, GSE77753), (c) a group with vascular EDS (vEDS) (31), PMID: 29346445, GSE102042), and (d) a cohort of patients suffering from classical EDS (cEDS) (32), PMID: 30716086, GSE117680), which carry distinct but overlapping symptoms with spEDS. Results present the DEG overlap and uniqueness between the 5 transcriptomes (Figure 5E). There were no genes common to the 5 studies, and only 11.2% (247 of 2,194) of genes overlapping between at least 2 experiments, indicating that each transcriptome is highly specific to the different subtypes of pathologies, concordant with the fact that they originate from separate molecular defects. Twelve genes were shared between our study and the one published by Ritelli et al. (29), which include patients carrying a p.(R256W) and the frameshift p.(I76Thrfs*202) mutations in β3GalT6. The 12 genes also included *ARHGEF3* (involved in bone biology), *HOXD10* and *HOXD11* (limb development), CSRP2 (vascular system), *NLGN1* and *GRIK2* (nervous system), all involved in various aspects of the developmental process. In addition, *SULF1,* coding for Sulf1, is involved in HS-GAG chains maturation and was also dysregulated in JHS/EDS-HT, which may affect cell signaling, depending on heparin-binding growth factors. Altogether, our analysis reveals a transcriptomic signature specific for patients with *B3GALT6*-spEDS and suggests that collagen maturation is an essential dysregulated process along with several altered developmental pathways.

### B3galt6-deficiency impairs Col XII GAG substitution and alters ECM structure and mechanical properties.

**Figure 6:**
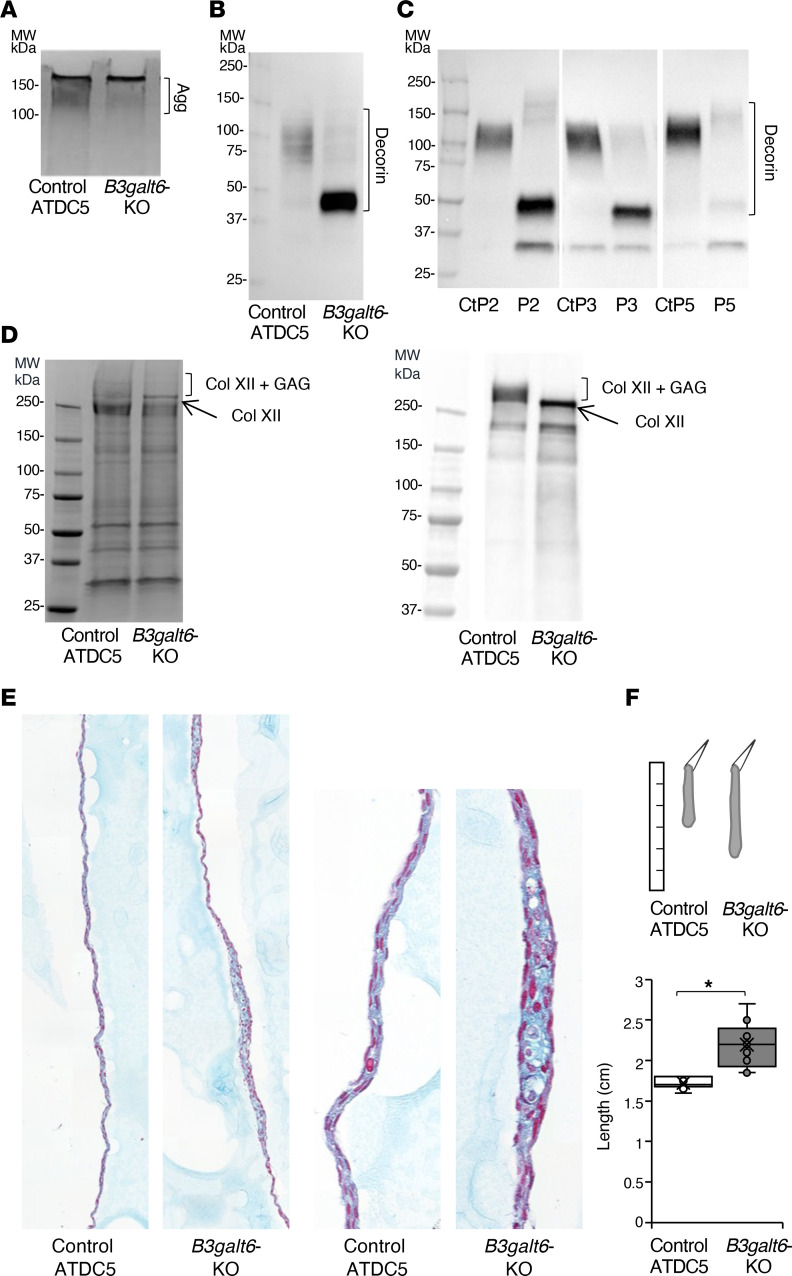
*B3galt6* invalidation in ATDC5 cells reveals defective glycosylation of Col XII and matrix alterations. (**A**) Representative image of the large PGs (mostly aggrecan, Agg) using Alcian Blue staining (*n* = 3 pseudotissues). (**B**) Representative immunoblot of decorin in control and *B3galt6*-KO pseudotissues (*n* = 3 independent experiments). (**C**) Representative immunoblot of decorin in control and spEDS patient fibroblasts treated with ascorbic acid. CtP2, CtP3, and CtP5 are the respective controls for patients P2, P3, and P5 (*n* = 3 independent experiments). (**D**) Representative protein profile of guanidine extracts (left panel) and immunoblot analysis of Col XII in pseudotissues (right panel) (*n* = 3 pseudotissues). (**E**) Histological staining of pseudotissues with Alcian Blue. Images were obtained with optical microscope at 10× (left) and 60× (right) magnification. Images are representative of staining on 3 pseudotissues. (**F**) Illustration of gravity assay on pseudotissues (upper panel). Length of the pseudotissues (lower panel). Data are presented as box plot with the mean symbolized by an X (*n* = 3 independent experiments); *P < 0.05 (Student’s *t* test).

We used the ATDC5 chondrogenic cell line as a 3D model of matrix synthesis to investigate the interplay between GAGs, collagens, and ECM defects (33, 34). Following *B3galt6* invalidation, ATDC5 cells were grown in chondrogenic media to allow matrix synthesis, resulting in the formation of a coherent structure referred to as a “pseudotissue.” Guanidine extracts (corresponding to noncrosslinked ECM components, essentially PGs and glycoproteins) were prepared from this material. In Figure 6A, SDS-PAGE analysis of this fraction showed a reduced abundance of GAG chains from large PGs (mainly aggrecan, as previously identified by mass spectrometry [MS]) (33). Glycosylation of endogenous decorin was also barely detectable in the *B3galt6-*KO cells (Figure 6B). Similarly, the glycosylation of decorin in spEDS fibroblasts was dramatically reduced compared with control individuals (Figure 6C). It is noteworthy that the size of the remaining CS/DS chains was increased in patients P2 and P5, but not in P3.

Analysis of the guanidine extracts from the pseudotissues revealed an additional band (> 250 kDa) in the *B3galt6*-KO cells (Figure 6D). The band was identified as the Col XII, also known as a PG, through MS analysis. Immunoblot analysis showed that the control ATDC5 cells expressed the GAG-substituted long form, visible as a smeary band more than 250 kDa, and possibly the short form (devoid of GAG-glycosylation sites) (Figure 6D). In *B3galt6*-KO cells, immunoblot analysis showed the presence of a sharp band corresponding to the long form of Col XII without GAG chain(s) and possibly the short form. It is noteworthy that analysis of the cyanogen bromide (CnBr) extracts that contain fibrillar and cross-linked collagens didn’t reveal obvious changes in the electrophoretic profile (Supplemental Figure 11).

Histological analyses show that the surface of the *B3galt6*-KO pseudotissue was irregular and contained a less coherent matrix, suggesting an alteration of ECM integrity upon gene KO (Figure 6E).

Gravity assays showed a significant increase in extensibility (~ 27%) of the *B3galt6*-KO pseudotissue compared with the control (Figure 6F), indicating a disturbed mechanical behavior. Overall, *B3galt6* invalidation resulted in significant GAG defects, including Col XII, that could be associated with critical structural and mechanical disturbances of the ECM during spEDS.

### Atomic Force Microscopy analyses reveal altered ECM biomechanical properties upon B3galt6-KO in ATDC5 cells.

**Figure 7:**
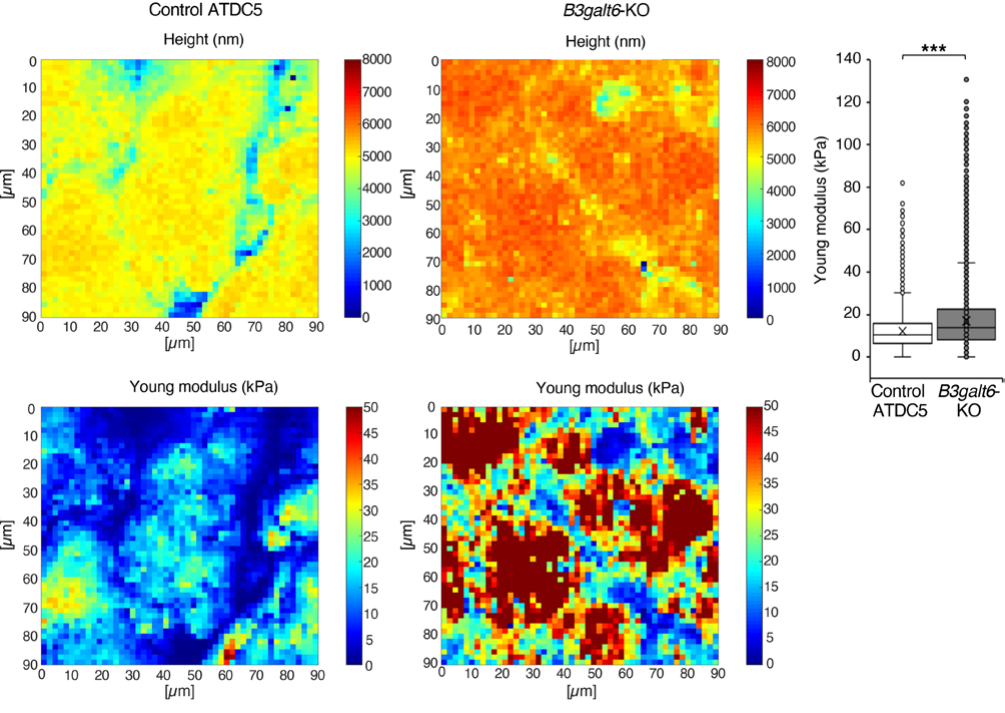
*B3galt6* invalidation in ATDC5 cells reveal alterations in ECM nanomechanical properties. Representative maps of the height (in nm) and the Young’s modulus (in kPa) of control and *B3galt6*-KO pseudotissues. Distribution of Young’s moduli in control and *B3galt6-KO* pseudotissues (left panel). Data are presented as box plot with the mean symbolized by an X (measurements on at least 5 pseudotissues); ***P *<* 0.001 (Student’s *t* test).

To assess the impact of impaired GAG-collagen interactions on the ECM, we performed atomic force microscopy (AFM) experiments on control and *B3galt6*-KO pseudotissues. Height and stiffness of the pseudotissues were assessed using nanoindentation (Figure 7). Spatial mapping of tissue height indicated that control pseudotissue exhibited lower values than *B3galt6*-KO, for which a higher range of values was observed. Concomitantly, stiffness mapping indicated that *B3galt6-KO* pseudotissues exhibited significantly higher Young’s moduli (median 13.95 kPa) than control (10.50 kPa), despite large variations within a same pseudotissue (Figure 7). Indeed, both pseudotissues presented heterogenous distribution of Young’s moduli with stiffer zones corresponding to higher heights, that probably correspond to cell nuclei. These observations are concordant with the composition of the pseudotissues, which consist of differentiated ATDC5 cells surrounded by fibrillar collagens and noncollagenous proteins, namely PGs and glycoproteins. Altogether, our data suggested that the *B3galt6*-KO pseudotissue presented large variations in Young’s moduli and higher tissue stiffness compared with control, due to the loss of PG-GAGs and subsequent changes in collagen microarchitecture due to their absence.

## Discussion

*B3GALT6*-spEDS is the most prevalent genetic linkeropathy with approximately 50 pathogenic variants reported since the discovery of the first mutations (14, 15). Despite promising progress in the description of its clinical presentation, the underlying mechanisms of *B3GALT6-*spEDS are poorly understood. The integrated molecular and cellular strategies described here fill important gaps in our understanding of *B3GALT6-*spEDS pathobiology. Specifically, our study illuminates the mechanisms by which gene mutations affect β3GalT6 function and the subsequent synthesis of GAGs. We demonstrate how these defects, despite the presence of a residual GAG synthesis, impair collagen maturation and ECM biomechanics.

At the molecular level, we report the efficient expression and purification of a truncated form of β3GalT6 in bacteria, fully active against the acceptor substrate Gal-Xyl(2P)-OMN. We found that the 3 mutations Y182C, D207H, and G217S result in a complete LOF of the recombinant enzyme, which is concordant with the reduced galactosyltransferase activity in patient fibroblasts carrying these mutations. Nakajima et al. (15) evaluated the activity of the following mutations, p.(M1?), p.(S65G), p.(P67L), p.(D156N), p.(R232C), and p.(C300S) and p.(S309T), identified in patients with spEDS and SEMD-JL1 and found that all expressed mutated enzymes, except p.(R232C), presented very low galactosyltransferase activity toward Gal-*O*-naphthyl. These results from our lab and others underscore dramatic effects of the pathogenic variants on enzyme activity.

The β3GalT6 belongs to the GT31 family of the CAZy database (http://www.cazy.org/), and its 3D structure has not been solved yet. To overcome this lack of information, we performed large-scale atomistic MD simulations to explore the conformational dynamics of β3GalT6, revealing a common mechanism accounting for the deleterious effect of the selected mutations. Comparison of the interaction network between WT and the 3 mutants highlights the loss of a salt bridge between K193 and D242. These 2 residues are located in a long flexible loop (G188-P211) and within the α8 helix of the catalytic domain, respectively. We propose that the K193-D242 salt bridge plays a role in stabilizing the active site of β3GalT6 by connecting key elements that belong to the catalytic center and possibly allow interactions with the substrates. Indeed, analysis of the 3D structure of B3GNT2 (35), another member of the GT31 family, shows that the corresponding loop (F275-G307) contains important residues involved in donor substrate binding. In B3GNT2, K288 and Y289 form hydrogen bonds with the pyrophosphate of UDP. As these amino acids are replaced by conservative substitutions in β3GalT6 (R197 and W198, respectively), they are anticipated to fulfill equivalent roles (Supplemental Figure 3C). Similarly, Leoni et al. (36), based on homology modeling using B3GNT2 as template, predicted that the p.(R197C) mutation, causing a severe syndrome, would substantially alter the UDP-Mn^2+^ binding site and catalytic activity of β3GalT6. Our MD simulations add further information by showing that, in β3GalT6, the P194-C205 segment to which R197 and W198 belong, shows high fluctuations, and that the flexibility of this domain is reduced in all mutants, reinforcing the idea that disturbance of the UDP binding site is an important factor contributing to the observed LOF. This is supported by the identification of a p.(C206W) mutation causing early lethality of a child with Al-Gazali syndrome, which was attributed to major conformational changes of the enzyme (22). In the B3GNT2 structure, the F275–G307 loop also contains residues interacting with the acceptor substrate. However, since the substrate differs from that of β3GalT6, further study is needed to confirm whether the corresponding loop in β3GalT6 has a similar role. In all 3 mutants, the missing salt bridge involves D242, a residue belonging to a conserved ×ED motif, predicted to be the catalytic base. As described for D333 in B3GNT2, and D255 in core 1/T-synthase C1GalT1 (37), another CAZY31 enzyme, it could facilitate the nucleophilic attack of the acceptor Gal*O*3 hydroxyle onto the anomeric *C*1 of the Gal donor substrate, a mechanism deployed by most inverting GTs (38). Overall, the loss of a link between 2 critical elements for the catalytic domain provides a convincing rationale for the LOF observed in the 3 mutants. This hypothesis, based on predictive methods, needs to be confirmed by structural analysis.

Next, we determined the impact of the β3GalT6 LOF on GAG synthesis. We showed previously that the capacity of patient fibroblasts to prime GAG synthesis using 4-MUX was severely compromised, but not absent (14, 16). In our study, similarly, the CS/DS chain of decorin was still detectable and, noticeably, its length was increased. In addition, previous studies revealed reduced (but detectable) HS expression on the cell membrane of patient fibroblasts (14, 16), and in patient lymphoblastoid cells (15). Collectively, these data indicate that GAG chain synthesis is not totally abrogated in *B3GALT6-*spEDS patient cells.

In *B3GALT6*-KO H7 cells, the levels of endogenous GAGs and radiolabeled decorin were greatly reduced for the H7-D207H and H7-G217S mutants, highlighting their deleterious effect in cellulo. In contrast, GAG synthesis in these mutants, as well as in mock-transfected cells, was restored to levels similar to those in H7-WT cells with 4-MUX (5 μM). Furthermore, we showed that, despite the lack of enzymatic activity in vitro, H7-Y182C cells produce GAG chains at levels similar to WT, as previously described for patient VII.1 (16). The higher capacity of H7-Y182C cells to maintain GAG synthesis can be explained by the fact that the enzyme is expressed as a transmembrane protein (while the purified protein is truncated) and by the distinct positioning of Y182, and the 2 residues, D207 and G217, which are located within and on, respectively, the other side of the flexible loop. It also suggests that Y182 is more likely to play a role in the structure than in catalyzing or binding the donor substrate. Our findings are concordant with Chen et al. (39), who reported that Chinese Hamster Ovary (CHO) *B3galt6*-KO cells retained the capacity to produce GAGs, while the corresponding *Xylt2*, *B4galt7*, or *B3gat3*-KO cells were incapable of doing so, suggesting a peculiar mechanism when β3GalT6 is deficient.

These results prompted us to explore the formation of an alternate linkage GlcA-Gal-Xyl serving as a GAG primer. Supporting this proposal, Persson et al. (40) described a noncanonical linkage region in bikunin from urine of healthy individuals, and in CS chains primed from xylosides in human cell lines. In line, in a *B3galt6*-KO zebrafish model, Delbaere et al. (27) showed that the PGs possessed a trisaccharide linkage region. This was confirmed by the analysis of the GAG linkage region of bikunin from patients with *B3GALT6-*spEDS (26). Our results show that decorin glycosylation was further reduced after *B3GAT3* knockdown in spEDS fibroblasts and indicate that GlcAT-I would act after β4GalT7 when β3GalT6 activity is absent or very low.

In agreement with the previous results, we showed that the recombinant GlcAT-I was able to use a Gal-Xyl analogue and also to catalyze the formation of the trisaccharide linker when incubated with a small peptide mimicking the CSF1 PG. In contrast, when GlcAT-I was overexpressed in cellulo, the GAG synthesis capacity appeared to differ depending on the nature and/or availability of the acceptor substrate, and, potentially, on the level of expression of β3GalT6. This observation is in line with a previous study on *B4galt7-*KO CHO cells (41), which reported a possible different compartmentalization of exogenous xylosides compared with endogenous xylosylated core proteins, which could therefore facilitate or prevent GAG assembly depending on the cellular context. Further experiments, including coexpression of WT or mutant β3GalT6 and GlcAT-I are necessary to fully dissect the molecular mechanisms underlying this alternative process. Collectively, it appears that a pathogenic context promotes the formation of GAGs with an immature linkage region. However, this residual GAG synthesis seems to depend on the cellular environment and could involve GlcAT-I in its fine-tuning.

By transcriptome-wide expression profiling of spEDS fibroblasts, we identified several DEGs and overrepresented biological processes linked to collagen maturation and collagen fibrils assembly, which is consistent with abnormal collagen fibril architecture of skin biopsy from the patients with spEDS (14).

Previous studies document the major role of matrix PGs in regulating collagen organization and ECM behavior, even if the contribution of the GAG part is not often specified and controversial (42). SLRPs, such as decorin, play a role by either juxtaposing collagen monomers through steric hindrance or directly connecting them during fibril growth. (43). Decorin connects fibrillar collagens with minor collagens such as collagen VI (44) and it also interacts with FACITs, such as collagens XII and XIV, which interconnect collagen fibrils (45). Additionally, large CS-PGs, such as versican and aggrecan, also regulate collagen behavior in a distinct and potentially opposite manner to SLRPs (42). Therefore, the strong defects in CS chains observed in our study are likely to severely affect collagen organization.

It is noteworthy that the most strongly DEGs identified in our study were *LOXL1*, *LOXL2*, and *LOX,* coding for 3 LOX family members, which catalyze the oxidative deamination of specific lysine residues within collagens, causing covalent collagen cross linking (46). The transcriptomic spEDS signature also includes *PLOD1*, coding for the lysyl hydroxylase LH1, which modifies a subset of lysine residues within the triple-helical region of collagen, thereby modulating the structure and valency of collagen cross links (47). Notably, mutation of this gene causes the kyphoscoliotic type of EDS (kEDS), which shows major clinical overlap with spEDS (48). However, it is surprising that only 12 genes overlap between the 2 transcriptomic analyses of patients deficient in *B3GALT6*. This may be explained by the fact that Ritelli’s study is based on 2 sisters, who have considerably less genetic variation than unrelated individuals. Moreover, these sisters are affected by a SEMD-JL1-type syndrome, whereas, in our work, only one patient (VII.1) was diagnosed with this disease (16). Collectively, transcriptome profiling reveals changes in collagen organization and alterations in critical developmental pathways that are likely to explain the perturbation of physiological functions, from early morphogenesis to postnatal development.

We showed that, among other PGs, the GAG substitution of the FACIT Col XII was severely impaired. Widely expressed in CTs, Col XII regulates collagen fibril spacing and assembly, as well as cellular function during tissue development and regeneration (49). It is a collagen homotrimer of 3 a1(XII) chains with 2 major alternatively spliced variants, the large variant carrying four predicted GAG attachment sites (50). Col XII and its binding partners, including SLRPs and tenascin X, form flexible bridges between collagen fibrils and other noncollagenous molecules, and thus regulate the organization and mechanical properties of collagen fibrils in various tissues (45). Mutations in *COL12A1* cause myopathic EDS (mEDS), the symptoms of which overlap with those of spEDS. However, the function of the Col XII GAG chains is poorly documented. The defective GAG substitution of Col XII discovered here may be relevant to the pathogenesis of spEDS.

In our work, higher Young’s modulus median value was calculated in *B3galt6-*KO pseudotissues using AFM-nanoindentation technique. The nanomechanical changes within our *B3galt6-*KO model are likely to be due to the combination of at least 2 mechanisms, including the loss of water retention capacity, directly caused by the reduced GAG content, and a disorganized FACIT-collagen network resulting from impaired PG-GAG–collagen interactions. Interestingly, Vaez et al. (51) showed that glycation of collagen fibrils in collagen scaffolds leads to a higher Young’s modulus, presumably due to changes in collagen crosslinks and higher water sorption capacity. This highlights the importance of GAG negative charges and water content in the biomechanical properties of CTs. In *Col12a1^–/–^* mice, the absence of Col XII led to disrupted collagen organization and increased stiffness of the corneal stroma as assessed by AFM (52). Similarly, the increased tissue stiffness observed in our study could be due, at least partially, to the defective Col XII glycosylation. Notably, several studies have attempted to establish functional relationships between collagen organization and stiffness. In general, tissues from patients with EDS have lower elastic modulus and lower ultimate strength, which have been tentatively associated with EDS symptoms (53). In an ECM model produced by skin fibroblasts, ECM stiffness in *PLOD1*-deficient cells from patients with kEDS was slightly but not significantly lower than controls (54). In our study, greater surface stiffness was shown in *B3galt6*-KO pseudotissues, suggesting that our ECM model recapitulates the characteristics of the matrix environment. It is therefore a relevant model for studying CT disorders by assessing the function of collagen, GAG-PG, and matrix bridging molecules as a whole. One limitation to our biomechanical studies is the need to better delineate the regions within the pseudotissue to directly correlate the biomechanical measurements with the biochemical and molecular modifications.

In summary, we describe here an integrated multifaceted approach that uncovers the consecutive mechanisms triggered by *B3GALT6* mutations, from protein LOF to the altered biomechanical properties of CTs in spEDS. Our study highlights the importance of better understanding the functional and physical interactions occurring within the ECM and reveals a new link between GAG synthesis and collagen maturation, implicating Col XII. Our study provides evidence for the development of new therapeutic strategies to stimulate the GAG chain biosynthetic pathway and limit the deleterious effect of GAG deficiency, thereby improving the health and quality of life of patients.

## Methods

### Sex as a biological variable

Patients included in our study were selected based on the type of *B3GALT6* mutation, regardless of their sex and age. We do not expect the results to be impacted by age or sex.

### Materials

Cell media, supplements, and reagents were purchased from Sigma-Aldrich unless stated otherwise. The synthetic compounds Gal-Xyl-OMN, Gal-Xyl(2P)-OMN, Gal-Gal-Xyl-OMN and Gal-Gal-Xyl(2P)-OMN were synthesized as previously described (55). The UDP-Gal was provided by Carbosynth.

#### Cloning, expression and purification of MBP-β3GalT6 and GST-GlcAT-I fusion proteins in E. coli.

Two truncated constructs of the human β3GalT6 lacking either the first 29 N_ter_ (MBP-ΔN_ter_29-WT) or 50 N_ter_ (MBP-ΔN_ter_50-WT) amino acids were designed and cloned into the pETM41 expression vector (Thermo Fisher Scientific) containing a N_ter_ 6xHis-tag and a MBP fusion partner, followed by the sequence of interest. The β3GalT6 sequence was amplified by PCR from human placenta QUICK-Clone cDNA (BD Biosciences Clontech) using appropriate primers, then inserted into the pETM41 expression vector using NcoI (5′ end) and XhoI (3′ end) restriction sites (Supplemental Table 2). The 2 constructs were sequenced and transformed in *E*. *coli* Rosetta2 (DE3). Cells were grown at 37°C in 1 L LB broth supplemented with kanamycin (100 μg/mL) until an absorbance of 0.6 at 600 nm. Isopropyl β-D-1-thiogalactopyranoside (IPTG) was added to the culture at 1 mM final concentration and incubated overnight at 20°C. Cells were harvested and washed in 50 mL PBS by centrifugation at 5,000*g* for 10 minutes. Pellets were resuspended in 30 mL of MBP lysis buffer (20 mM Tris-HCl, pH 7.4 containing 200 mM NaCl, 5% glycerol, and 1 mM DTT) and disrupted by sonication (8 × 1 minute bursts separated by 1 minute of ice cooling). The lysate was centrifuged at 13,000*g* for 20 minutes at 4°C. The supernatant was treated for 1 minute at room temperature using benzonase (Merck) and filtrated through a 0.45 μm filter.

All purification steps were performed at 4°C. A 1 mL MBPTrap column (Cytiva) was equilibrated with 10 column volumes of MBP binding buffer (20 mM Tris-HCl, pH 7.4 containing 200 mM NaCl and 5% glycerol). The supernatant was loaded onto the column and then washed with 10 column volumes of wash buffer (20 mM Tris-HCl, pH 7.4 containing 300 mM NaCl and 5% glycerol). Bound proteins were eluted with elution buffer containing 10 mM of maltose. Fractions containing the protein of interest were pooled and concentrated. The purity of the collected fractions was assessed by SDS-PAGE and Coomassie blue staining.

The mutant constructs MBP-ΔN_ter_29-Y182C, MBP-ΔN_ter_29-D207H and MBP-ΔN_ter_29-G217S were obtained from pETM41-β3GalT6ΔN_ter_29-WT and the appropriate primers (Supplemental Table 2) using the QuikChange site-directed mutagenesis kit (Agilent Technologies France). Mutations were verified by sequencing.

The cloning, expression and purification of GST-GlcAT-I fusion protein in *E*. *coli* were described previously (56).

### GT assays

The galactosyltransferase assays involved incubating 50 pmol of purified β3GalT6 in 50 mM MES buffer (pH 6.5) containing 100 mM NaCl and 10 mM MnCl_2_ and the appropriate concentrations of substrates during 60 minutes at 37°C. To determine the kinetic parameters, various concentrations of Gal-Xyl(2P)-OMN (0–5 mM with 10 mM UDP-Gal) or UDP-Gal (0–10 mM with 5 mM Gal-Xyl(2P)-OMN) were used. Reactions were stopped as previously described (57) and analyzed by HPLC on a reverse phase XBridge C18 column (150 × 5 mm, 4 μm) (Waters SAS) linked to a e2695 Separation Module (Waters) and a e2475 Multiwavelength Fluorescence Detector (Waters) (Excitation wavelength = 324 nm; detection wavelength = 356 nm). The mobile phase was composed of 11% acetonitrile and 0.02% trifluoroacetic acid. Kinetic parameters were estimated by fitting data by nonlinear regression to the Michaelis-Menten model (GraphPad Prism 7).

The glucuronosyltransferase assays involved incubating 100 pmol of purified GlcAT-I in 100 mM sodium acetate buffer (pH 6.5) containing 10 mM MnCl_2_ and 5 mM UDP-GlcA in the presence of appropriate substrates during 60 min at 37°C. Then, GlcAT-I activity was evaluated using UDP-Glo glycosyltransferase assay kit (Promega).

In vitro enzymatic synthesis of the linker region was performed by the stepwise addition of the sugar moieties using recombinant GTs and a fluorescently labeled 8-amino-acid peptide mimicking the CSF1 PG CS attachment as previously described (56).

### Circular dichroism

Far-UV CD spectra of WT or mutant β3GalT6 (20 μM) were recorded on a Chirascan Plus spectrometer (Applied Photophysics) at 25°C using a 0.01 cm pathlength quartz cell in a 10 mM sodium phosphate, 100 mM sodium fluoride, pH 7.4 buffer. Measurements were done at 1 nm/sec between 180 and 260 nm. Data were exploited after removal of background signal from the buffer alone.

### Structural modeling of human β3GalT6

The AlphaFold2 model of human β3GalT6 was used (UniProtKB: Q96L58). The globular domain of this model was close to the TM domain, which could pose potential atomic clashes with the membrane during MD simulations. To avoid this, we used Rosetta relax protocol (58) which refines the structure based on local conformational space (no extensive refinement), generated 100 models, and picked the one with maximum distance to the membrane.

### MD simulations of human β3GalT6 and mutants

#### System preparation.

The structure consists of 329 amino acids including a N_ter_ Ct fragment, a TM segment, a stem region, and a catalytic domain. MD simulations of the β3GalT6 were conducted with the TM part inserted in a Golgi membrane (17% Cholesterol, 49% DOPC, 16% DOPE, 5% DOPS, 6% PSM, 6% POPI, and 1% PLPA). Histidine protonation states were assigned using MolProbity. The WT and 3 mutants (Y182C, D207H, and G217S) were built with CHARMM-GUI (59) - membrane builder (http://www.charmm-gui.org/?doc-1?4input/membrane) and CHARMM36m force field, solvated with TIP3P water (12Å buffer) and 150 mM NaCl. Each system underwent 3 1-μs replicates.

Production of the trajectories - All simulations were performed using GROMACS 2021.3 (60). Energy minimization was carried out with the steepest descent algorithm for 5,000 steps, followed by NPT equilibration at 310 K, allowing lipid and solvent relaxation around the restrained protein. Harmonic restraints were applied to all protein and lipid nonhydrogen atoms and gradually reduced over 6 steps, totaling 1.875 ns. Electrostatics were treated with the PME method and van der Waals interactions were force-switched off between 10–12Å. Production runs were conducted in the NPT ensemble with a 2.0 fs time step, 310 K temperature (Nosé-Hoover thermostat), and 1 atm pressure (Parrinello-Rahman barostat). The SHAKE algorithm was used to constrain bonds involving hydrogen atoms. Coordinates were saved every 10 ps.

#### Analysis of the trajectories.

Standard MD trajectory analyses were performed using the gmx module of GROMACS 2021.3. Backbone RMSD (Cα, C, N, O) relative to the initial frame was recorded for each replicate (Supplemental Figure 4A). Based on RMSD profiles, subsequent analyses were conducted over the last 900 ns, where systems were fully relaxed. Perresidue RMSF was computed for backbone atoms relative to the average structure (Supplemental Figure 4B). All systems remained stable during the simulations. Salt bridges were identified using VMD when the distance between oxygen atoms of acidic residues and nitrogen atoms of basic residues was less than or equal to 3.2Å in at least 1 frame. Their formation frequency was calculated as the number of frames where the center-of-mass distance between these atoms was less than or equal to 3.5Å, with the maximum value across 3 replicates recorded for each pair. Hydrogen bonds were detected using HBPLUS (61), applying geometric criteria: D–A ≤ 3.9Å, H–A ≤ 2.5Å, and angles ≥ 90° for D–H–A, H–A–AA, and D–A–AA (with AA indicating acceptor antecedent). For each H-bond between every pair of residues, strength was defined as the percentage of frames in which the bond was present, and the maximum value across replicates was assigned.

### Gene invalidation, HeLa cell culture and transfection

HeLa cells were cultured in DMEM with high glucose supplemented with 10% FBS, 1% glutamine, and 1% penicillin/streptomycin. *B3GALT6*-KO cells were generated by transfecting cells with pX458_sghB3GALT6-2 plasmid (TACGENE, CNRS UMR 7196-INSERM U1154-Sorbonne University) containing a single guide RNA (sgRNA) sequence (5′-ACGTGCTGCGGATCACGCTG-3′), the Cas9 gene and Enhanced Green Fluorescent Protein (EGFP) coding sequence. Forty-eight hours after transfection, cells were harvested and sorted using a cell sorter (Astrios, Beckman Coulter). One clone that had a 52-bp deletion (c.182_233del) in the *B3GALT6* gene leading to a frame-shift amino acid change followed by a stop codon at position 260 (mutant p.(V61Afs*199) (referred to as *B3GALT6*-KO H7 cells) (Supplemental Figure 5A) was selected. For transient transfection, *B3GALT6*-KO H7 cells were transfected using Turbofect (Thermo Fisher Scientific) with plasmids allowing the expression of WT, mutant β3GalT6-mCherry proteins (Sartorius Stedim) with optimized codon usage, or an empty vector. Media were refreshed on the following day. On the second day, cells were incubated overnight in Fisher’s medium containing 10 μCi/ml Na_2_[^35^SO_4_]) (Revvity SAS) and 4-MUX (2.5 or 5 mM) and processed as previously described for GAG analysis (57). The eluted GAGs were quantified by scintillation counting. Data were reported to DNA content by using the Quant-iT dsDNA HS Assay kit (Thermo Fisher Scientific).

In cotransfection experiments with pcDNA-decorin-His vector, cells were incubated overnight with serum-free medium or radiolabeled Fisher’s medium to analyze decorin expression. On the third day, decorin was precipitated from nonradiolabeled media for the immunoblot analysis or purified from radiolabeled media using Nickel magnetic beads. Radiolabeled decorin was resolved using Criterion Precast gels (4–15% Bis–Tris, Bio-Rad) and visualized by autoradiography using Amersham Hyperfilm MP (Cytiva).

### Patient fibroblast cell culture, GlcAT-I silencing, and treatment

Fibroblasts were isolated from skin biopsies obtained from age and sex-matched healthy individuals and spEDS patients and cultured as previously described (14, 16). For GlcAT-I silencing, spEDS fibroblasts were transfected with a siRNA targeting *B3GAT3* gene (s25257, Thermo Fisher Scientific) using DharmaFECT2 transfection reagent (Dharmacon), as previously described (11). Gene knockdown was verified using TaqMan gene expression assays for human *B3GAT3* (Hs01127629_m1, Thermo Fisher Scientific). The endogenous expression of decorin was assessed after treating skin fibroblasts with ascorbic acid for 14 days. In both experiments, decorin expression was assessed by immunoblot from conditioned media. Percentage of GAG-substituted decorin was calculated by measuring the intensities corresponding to the signal for the glycosylated form reported to the total signal (glycosylated form and core protein) using by ImageJ (62).

### Transcriptome analysis

Gene expression data are available in Gene Expression Omnibus and were generated following growth, extraction, labeling, hybridization, scan, and data processing procedures detailed for each sample under GEO accession GSE241724, as described previously (63). Hierarchical clustering heat maps were obtained on gene-median-centered data with uncentered correlation as a similarity metric. Volcano plots were rendered using EnhancedVolcano (Bioconductor). Disease gene signatures were obtained from the OpenTargets platform (64). Functional annotations were performed with EnrichR (65) for disease associations, gene ontology, and pathway enrichments in humans. For all experiments, *P* values < 0.001 or FDR < 0.05 indicated statistical significance, depending on the test availability and/or relevance.

For transcriptome comparison, transcriptomes were obtained from: this work (spEDS; 675 unique genes); Ritelli et al. (29) (heterozygous compound *B3GALT6* mutations from 2 sisters; GSE58312; 332 unique genes); Chiarelli et al. (30) (HT/JHS EDS; GSE77753; 206 unique genes); Chiarelli et al. (31) (vEDS; GSE102042; 700 unique genes); Chiarelli et al. (32) (cEDS; GSE117680; top 548 DEG = 548 unique genes). For validation of transcriptome data, total RNA was reverse transcribed into cDNA using iScript cDNA synthesis kit (Bio-Rad). Gene expression of *B3GALT6* (Hs00915402_s1), *LOXL1 (*Hs00935937_m1), and *LOXL2* (Hs00158757_m1) were measured using TaqMan gene expression assays (Applied Biosystems) and were normalized to *18S* (Hs99999901_s1).

### Generation of B3galt6-KO ATDC5 and cell culture

The ATDC5 cell line was purchased from the Riken Cell Bank (Tsukuba) and cultured in DMEM/Nutrient Mixture F-12 Ham supplemented with 5% FBS, 1% glutamine, 1% penicillin/streptomycin, 10 μg/mL of human transferrin and 3 × 10^–8^ M sodium selenite (33). ATDC5 cells were plated in 12-well plates for proteome analysis, and in 24-well plates onto glass coverslips and 13 mm calcium fluoride discs (Crystran Ltd) for histology and AFM analysis, respectively. Forty-eight hours after cell seeding, ATDC5 cells were inoculated overnight with 5 × 10^7^ recombinant adenoviral mammalian CRISPR-Cas9 vectors targeting mouse *B3galt6* (gRNA sequence: 5′ – GGTTCTCGTAGGCGTCGCGC – 3′) (VectorBuilder). Cell medium was refreshed daily for 3 days and then supplemented with insulin (10 μg/mL) for cell differentiation. After 14 days, cells formed a pseudotissue, which was either fixed using 4% PFA and stored in PBS buffer at 4°C or for gravity assay. In the latter case, the pseudotissue was detached and held with tweezers for 30 seconds while its length was measured with a ruler. RNA-seq analysis of control and *B3galt6*-KO ATDC5 confirmed that no *B3galt6* transcripts were detected following genome editing (Supplemental Figure 5A, unpublished data).

Protein fractionation on samples was performed as described in ref. 33 to obtain the secretome fraction (decorin), the guanidine extracts (Col XII, and CNBr extracts, containing soluble secreted proteins, noncross-linked and cross-linked matrisome, respectively. Protein fractions were resolved on 4–20% Protean TGX Stain-Free Gels (Bio-Rad) stained with Coomassie blue or transferred onto PVDF membrane for immunoblotting.

### Western blotting

The anti-human (MAB143) and the anti-mouse decorin (AF1060) and the anti-goat IgG (HAF017) antibodies were purchased from R&D Systems. The anti-Col XII (H-280, targeting amino acids 1861-2140) was purchased from Santa Cruz, the anti-mouse (#7076) and the anti-rabbit IgG (#7074) antibodies from Cell Signaling Technology (Ozyme) and the anti-HA antibody (H6908) from Sigma-Aldrich.

### Histological analysis of pseudotissues

Fixed pseudotissues were included into HistoGel (MM France) preparation before paraffin embedding. Transverse sections of tissue blocks were performed using Histocore Autocut R microtome (Leica Biosystems) and stained with Alcian Blue (bio-optica, MM France) and nuclear fast red (bio-optica) using Histocore Spectra stainer (Leica). Images were acquired using Lamina slide scanner (Akoya Biosciences, Perkin Elmer).

### Biomechanical analysis of pseudotissues

Nanomechanical analyses were performed on fixed pseudotissues in a liquid environment using a MFP3D-BIO AFM instrument (Asylum Research Technology, Oxford Instruments Company) using silicon nitride cantilevers (Bruker, MLCT) with a nominal spring constant of about 0.01 nN/nm^-–1^ at an indentation rate of 1 μm/s^–1^. At least 3 Force-Volume Images (FVI) at several different locations on the calcium fluoride disc were recorded for each condition. Each FVI consisted of a grid of 50 × 50 force curves measured adopting a 1 μm/s^–1^ approach rate of the tip toward the sample. The tissue stiffness (Young’s modulus) was evaluated by analyzing the force-indentation curves within the framework of the corrected Sneddon model (66, 67). All FVI were analyzed using an automatic MATLAB (The MathWorks Inc.) algorithm detailed elsewhere.

### Statistics

Statistical analysis was performed using Student’s *t* test to compare 2 groups and 1-way ANOVA test with Bonferroni post hoc correction for comparison among multiple groups.

### Study approval

This study was approved by the Ethics Committee of the Ghent University Hospital.

### Data availability

Data are available in the main text or the Supplemental Tables 3 and 4. Transcriptomic data were deposited in GEO under accession GSE241724. Values for all data points in graphs are reported in the Supporting Data Values file.

## Author contributions

RMD, BJ, JBV, SH, GF, YK, MB, AR, AW, DH, RO, SF, DS, and CB conducted experiments and acquired data. SF and CLB provided reagents. RMD, JBV, SH, GF, YK, HK, RW, NR, CLB, DS, FM, SG, CB, and SFG designed research studies and analyzed data. SFG and CB wrote and edited the manuscript; RMD, JBV, SH, GF, YK, RW, NR, GB, CLB, FM, and SG edited the manuscript.

## Supplementary Material

Supplemental data

Unedited blot and gel images

Supplemental table 3

Supplemental table 4

Supporting data values

## Figures and Tables

**Table 1:**
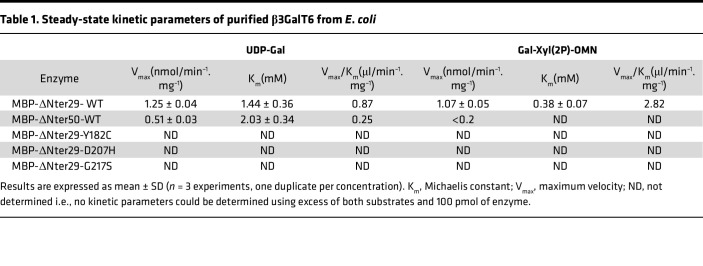
Steady-state kinetic parameters of purified β3GalT6 from *E*. *coli*

## Version 1 08/22/2025

Electronic publication

## Version 2 08/28/2025

Several characters that appeared in English were swapped with their Greek counterpart
