# Supplementary material for: B3GALT6 mutations lead to compromised connective tissue biomechanics in Ehlers-Danlos syndrome
Source: JCI Insight. 2025 Aug 22;10(16):e179474. doi: 10.1172/jci.insight.179474 (PMC12406734; doi:10.1172/jci.insight.179474)
Supplement: Supplemental data [file jciinsight-10-179474-s279.pdf]

Supplemental Figure 1

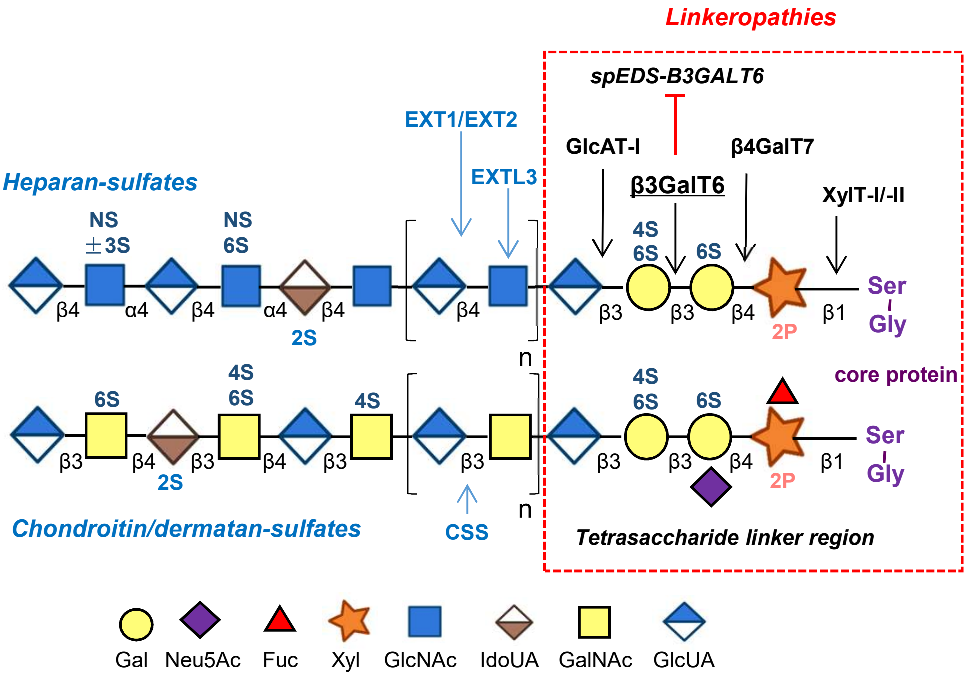

Figure 1. Schematic representation of GAG synthesis and associated "linkeropathies". From right to left, each saccharide residue is sequentially added onto the PG core-protein by a reaction catalyzed by a different GT. The GTs involved in the formation of the tetrasaccharide linker region and associated genetic diseases called "linkeropathies" are framed in a red dotted box. The linkeropathies associated with *B3GALT6* mutations include spEDS, EDS-like, spondyloepimetaphyseal dysplasia with joint laxity type 1 (SEMD-JL1) and Al-Gazali syndrome. XylT-I/-II, xylosyltransferase I/II; β4GalT7, β1,4-galactosyltransferase 7; β3GalT6, β1,3-galactosyltransferase 6; GlcAT-I, β1,3-glucuronosyltransferase 3; EXTL3, exotosin-like glycosyltransferase 3; EXT1, exotosin glycosyltransferase 1; EXT2, exotosin glycosyltransferase 2; CSS, chondroitin sulfate synthase; NS, N-deacetylated-N-sulfate; S, sulfate and the digit before S corresponds to the position of the sulfate group on the sugar ring; 2P, 2-O-phosphate; Xyl, xylose; Gal, galactose; GlcUA, glucuronic acid; GalNAc, *N*-acetylgalactosamine; IdoUA, iduronic acid; GlcNAc, *N*-acetylglucosamine; Fuc, fucose; Neu5Ac, *N*-acetylneuraminic acid.

Supplemental Figure 2

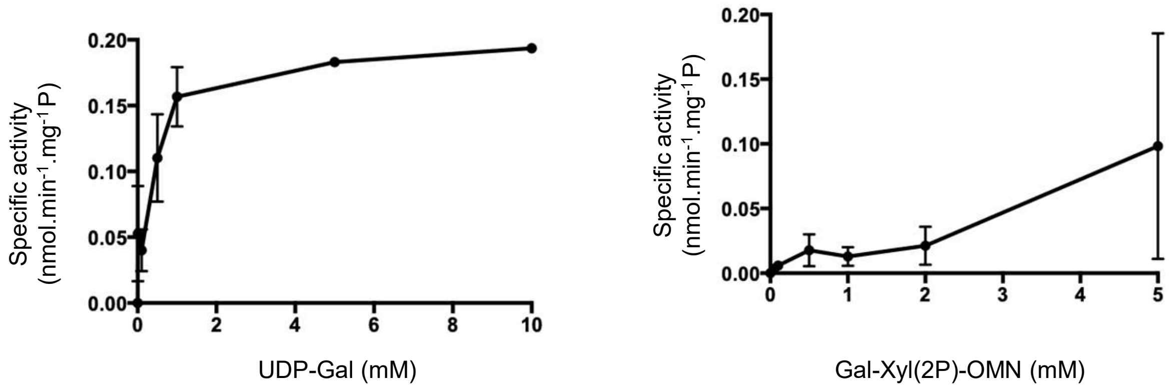

Figure 2. Kinetics of MBP-β3GalT6ΔN_{ter}50-WT towards the donor substrate UDP-Gal (left panel) and the acceptor substrate Gal-Xyl(2P)-OMN (right panel). Data are expressed as mean ± SD (n=3 experiments).

Supplemental Figure 3

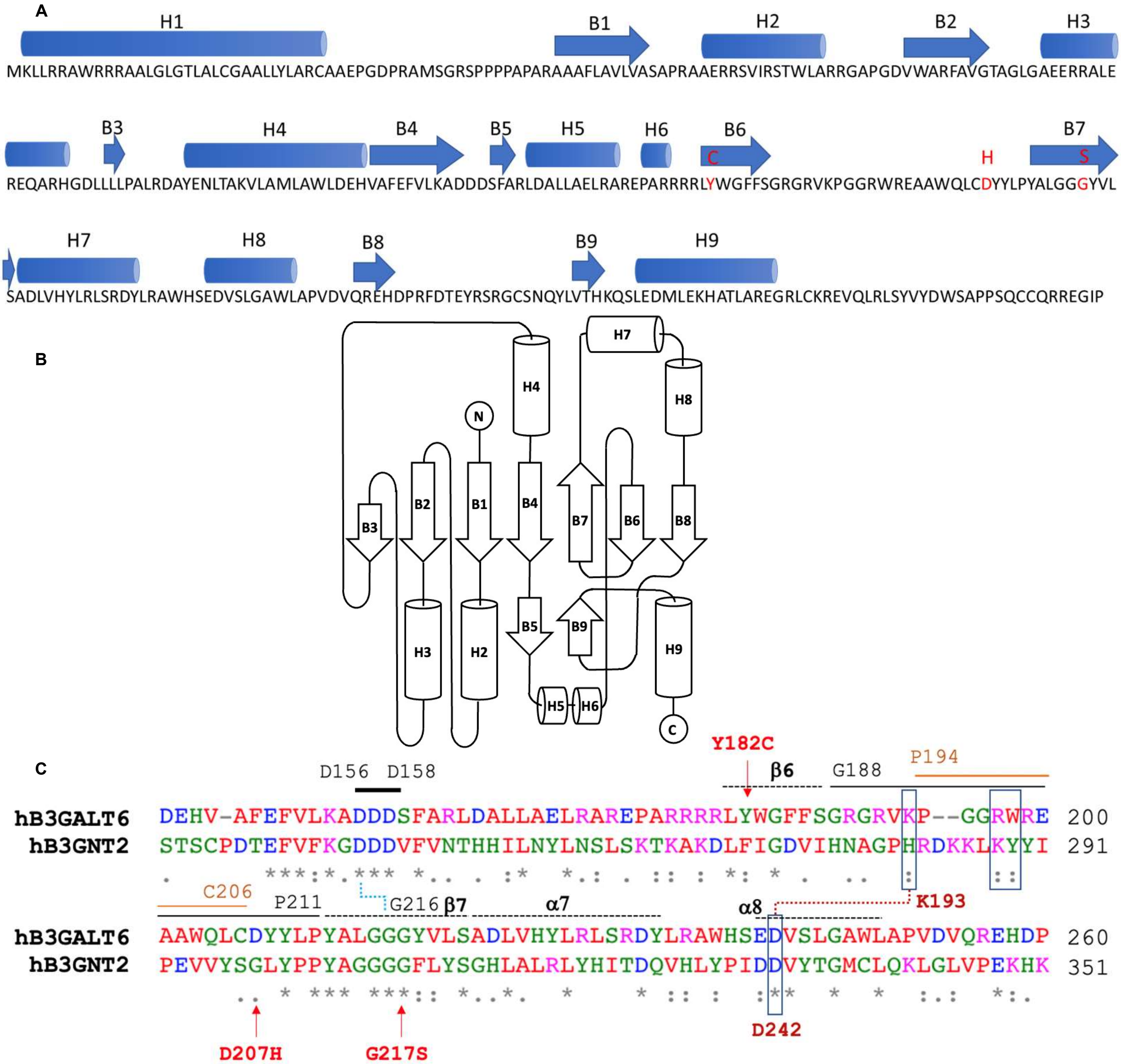

Figure 3. Sequence, structure of β3GalT6 and alignment with the human β1,3-*N*-acetylglucosaminyltransferase 2.
(**A**) Primary sequence and secondary structure of β3GalT6. Blue arrows and cylinders represent β strands (B) and α helices (H), respectively. Pathogenic variants p.(Y182C), p.(D207H) and p.(G217S) are indicated in red. (**B**) Topology diagram of β3GalT6. Arrows and cylinders represent β strands (B) and α helices (H), respectively. (**C**) Sequence alignment of the catalytic site of the human β3GalT6 (hB3GALT6, UniProtKB: Q96L58) and human β1,3-*N*-acetylglucosaminyltransferase 2 (hB3GNT2, UniProtKB: Q9NY97) using Clustal Omega software (29). Residues are coloured by the software according to their biochemical properties. Asterisks (*) indicate a fully conserved residue, colons (:) indicate strong similarity and periods (.) indicate weak similarity. β strands and α helices are indicated above the β3GalT6 sequence by black dotted lines. Cyan and carmine dotted lines indicate two important bonds of β3GalT6, respectively a hydrogen bond between D156 and G216 and a salt bridge between K193 and D242. Black full line indicates a long loop between G188 and P211 of β3GalT6, with an orange plain line indicating a high fluctuation domain within this loop. The DxD motif is represented by a black full and bold line. Red and bold annotations indicate the three point mutations of β3GalT6.

Supplemental Figure 4

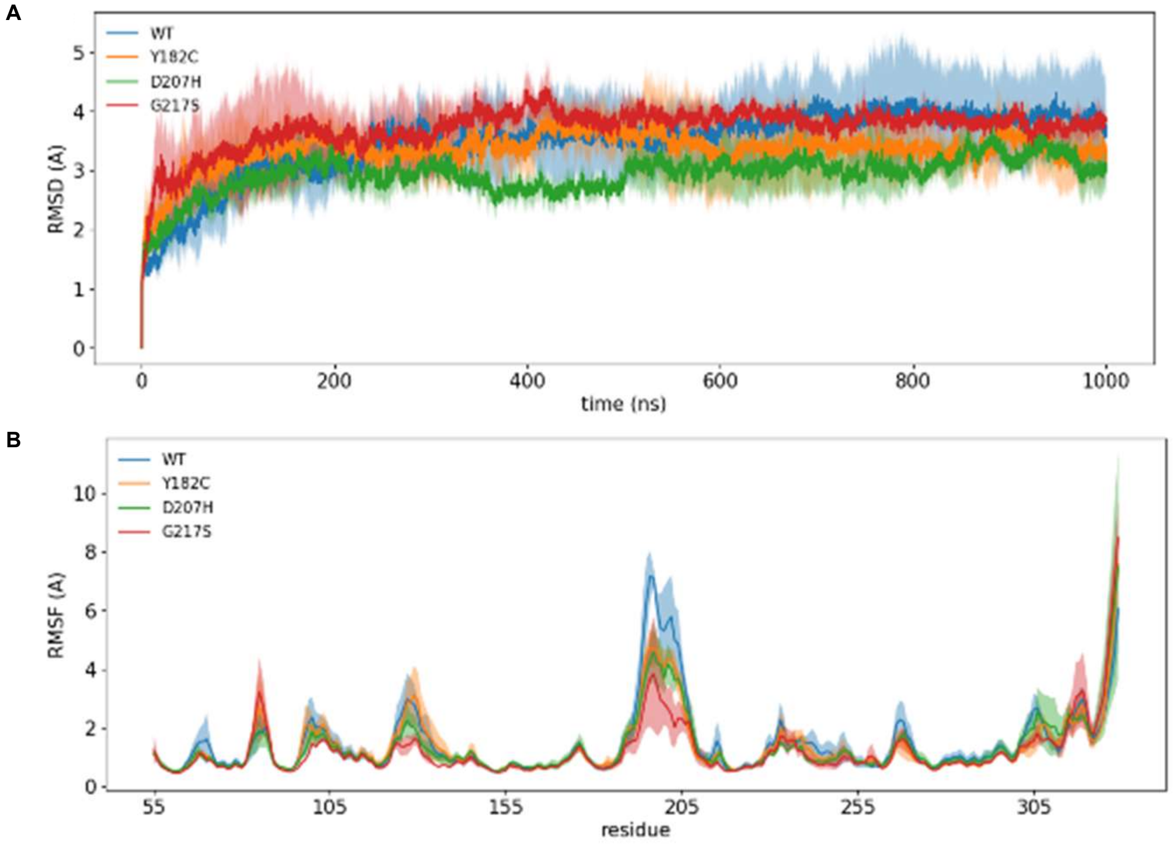

Figure 4. The average RMSD and RMSF of the studied systems from MD simulations. (**A**) The RMSD from the equilibrated structure was computed on the backbone (C, Ca, N, O) atoms and averaged over all the three replicates of WT and mutants. The average values are shown as lines and the shades correspond to the standard deviations. (**B**) The RMSF was measured on the backbone (C, Ca, N, O) atoms with respect to the average conformation and averaged by residue, considering the last 900 ns of the MD simulations. The values are averaged over the three replicates of each system. The average values are shown as lines and the shades correspond to the standard deviations.

Supplemental Figure 5

A

```
HeLa    GCCGCCGCCTTCCTGGCAGTGCTGGTGGCCAGCGCGCCCCGCGCCGCCGAGCGCCGCAGC
H7      GCCGCCGCCTTCCTGGCAG-----------------------------------------

HeLa    GTGATCCGCAGCACGTGGCTTGCGCGGCGCGGGGCCCCGGGCGACGTGTGGGCGCGCTTT
H7      -----------CACGTGGCTTGCGCGGCGCGGGGCCCCGGGCGACGTGTGGGCGCGCTTT

HeLa    GCCGTGGGCACGGCCGGCCTGGGCGCCGAGGAGCGGCGCGCCCTGGAGCGGGAGCAGGCG
H7      GCCGTGGGCACGGCCGGCCTGGGCGCCGAGGAGCGGCGCGCCCTGGAGCGGGAGCAGGCG

HeLa    CGGCACGGGGACCTGCTGCTGCTGCCCGCGCTGCGCGACGCCTACGAAAACCTCACGGCC
H7      CGGCACGGGGACCTGCTGCTGCTGCCCGCGCTGCGCGACGCCTACGAAAACCTCACGGCC

HeLa    AAGGTGCT
H7      AAGGTGCT
```

B

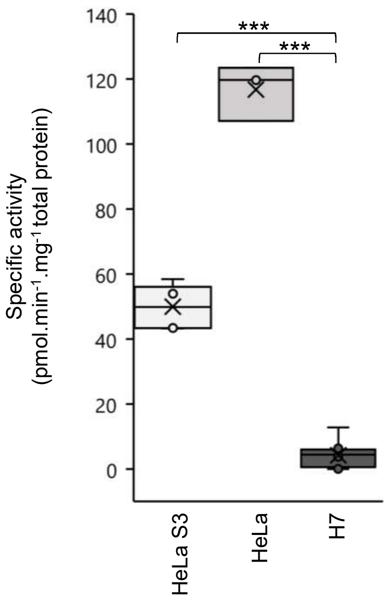

Figure 5. Invalidation of *B3GALT6* gene in *B3GALT6*-KO H7 cells and in vitro enzymatic activity of β3GalT6. (**A**) Sequence alignment of *B3GALT6*-KO H7 clone with HeLa cells (WT). Genomic DNA was extracted from *B3GALT6*-KO H7 and HeLa cells (WT) and the CRISPR-Cas9 target region was amplified by PCR. The PCR products were subcloned into a cloning vector and sent for sequencing. The alignment reveals a 52pb deletion in the *B3GALT6* sequence. The cDNA region including the sequence targeted by the single guide RNA was amplified by PCR and sequenced by Sanger method. Chromatograms of the PCR products from control and *B3galt6*-KO ATDC5 showed a heterogeneous profile near the PAM sequence, indicating the presence of indel events. (**B**) Analysis of in vitro β3GalT6 activity in total cell lysates from HeLa cells and *B3GALT6*-KO H7 cells. Enzymatic assays were performed using 1 mM acceptor substrate (Gal-Xyl(2P)-OMN), 5 mM donor substrate (UDP-Gal) and 10 µg total cell lysates. HeLa cells (2 cell lines) are considered as positive control. Data are presented as box plot with the mean symbolised by an X (n=2 experiments for HeLa S3, n=1 experiment for HeLa and n=3 experiments for H7, one triplicate per cell lysate); ***p<0.001 when compared to H7 clone (Student's t test).

Supplemental Figure 6

A

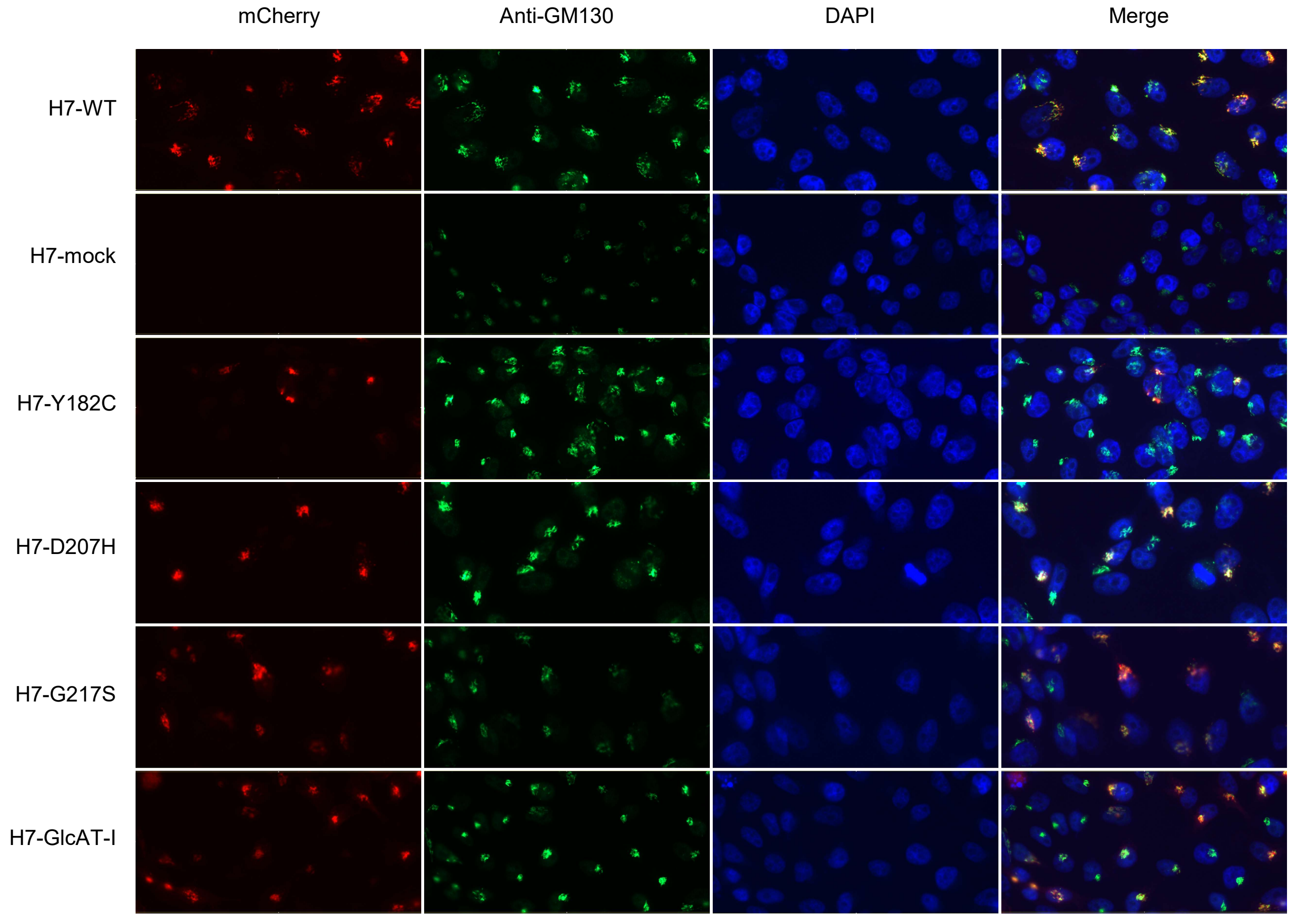

B

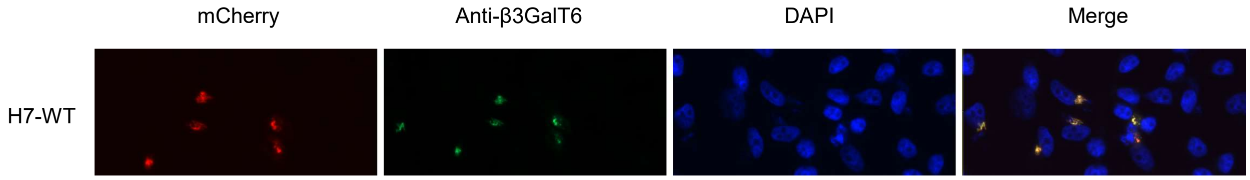

Figure 6. Representative image of the expression and localisation of WT and β3GalT6 mutants in *B3GALT6*-KO H7 cells. (**A**) *B3GALT6*-KO H7 cells were transiently transfected with plasmids allowing the expression of mCherry fusion proteins corresponding to WT, β3GalT6 mutants, GlcAT-I (Sartorius Stedim, Aubagne, France) or an empty vector (negative control). The GM130 cis-Golgi marker was used for protein co-localisation (**B**) Analysis of β3GalT6 expression and localisation using a commercial anti-β3GalT6 antibody. Nuclei were counterstained with DAPI (n=3 independent experiments).

Supplemental Figure 7

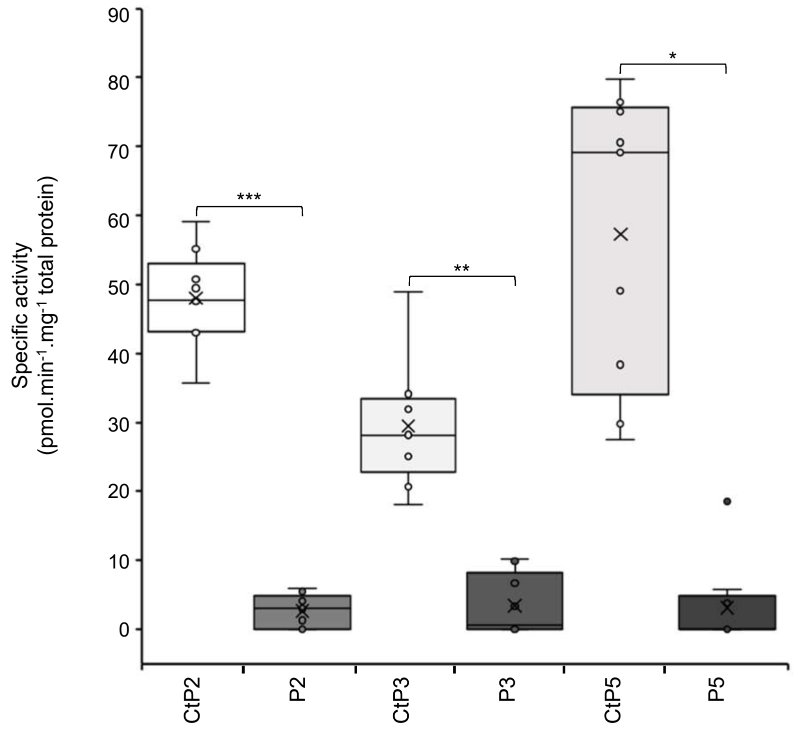

Figure 7. In vitro β3GalT6 activity in spEDS patient fibroblasts. β3GalT6 activity was measured using total cell lysates (10µg) prepared from control and patient fibroblasts. Gal-Xyl(2P)-OMN (2 mM) was used as acceptor substrate and UDP-Gal (1 mM) as donor substrate. Data are presented as box plot with the mean symbolised by an X (n=3 experiments; one triplicate per cell lysate); *p<0.5; **p<0.01; ***p<0.001 when P2, P3 and P5 were compared to CtP2, CtP3 and CtP5, respectively (Student's t test).

Supplemental Figure 8

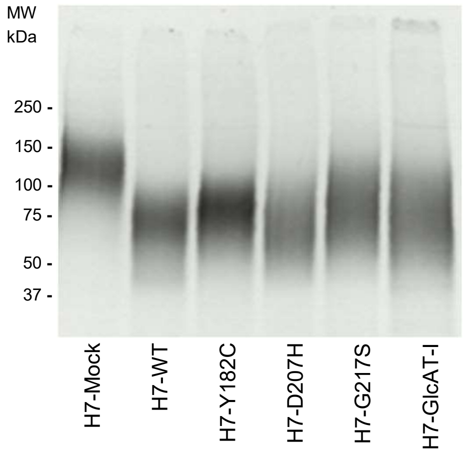

Figure 8. Representative profile of radiolabelled decorin in co-transfected *B3GALT6*-KO H7 cells. Radiolabelled decorin was secreted in conditioned media from *B3GALT6*-KO H7 cells transiently co-transfected with plasmids encoding WT, mutant β3GalT6, GlcAT-I or empty vector and decorin (n=3 experiments in triplicate). In order to visualize the profile of GAG residual synthesis, the same amount of radioactivity (8,000 dpm) was loaded in each lane.

Supplemental Figure 9

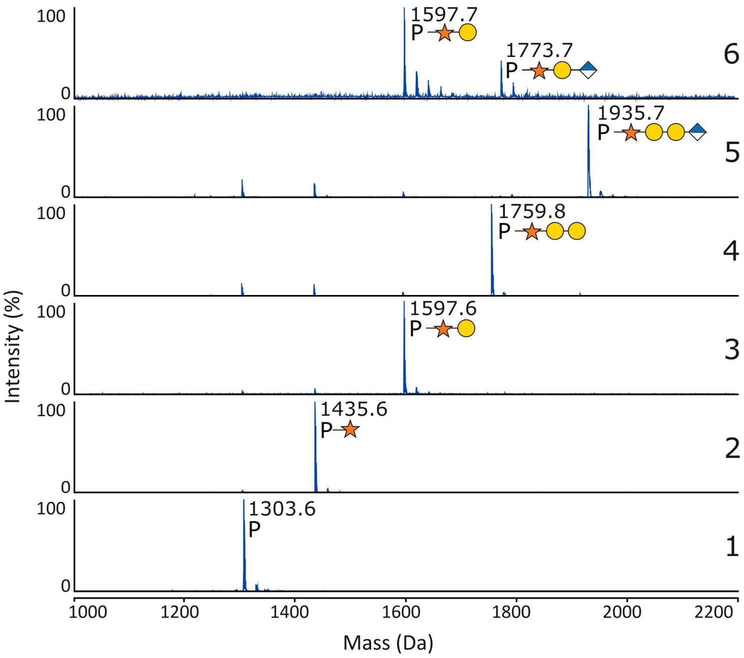

Figure 9. Analysis of reaction products by MALDI-MS. The composition and mass of the main peaks are indicated alongside. MALDI-MS determines the m/z (mass-to-charge) values of the products. For instance, the peptide 306EEASGEAS (referred to as P) has a mass of 1303.6 Da, and the corresponding peak of its singly charged ion has been detected at 1304.6 m/z. The addition of one xylose unit increases the mass by 132 Da (resulting in 1435.6 Da). Each galactose unit adds 162 Da (1597.6 and 1759.8 Da). One glucuronic acid unit further increases the mass by 176 Da (to 1935.7 Da). The major peak in reaction mixture 6 is a [Galβ1–4Xyl]-peptide (1597.7 Da), and the second-highest peak corresponds to a [GlcA-3Galβ1-4Xyl]-peptide (1773.7 Da).

Supplemental Figure 10

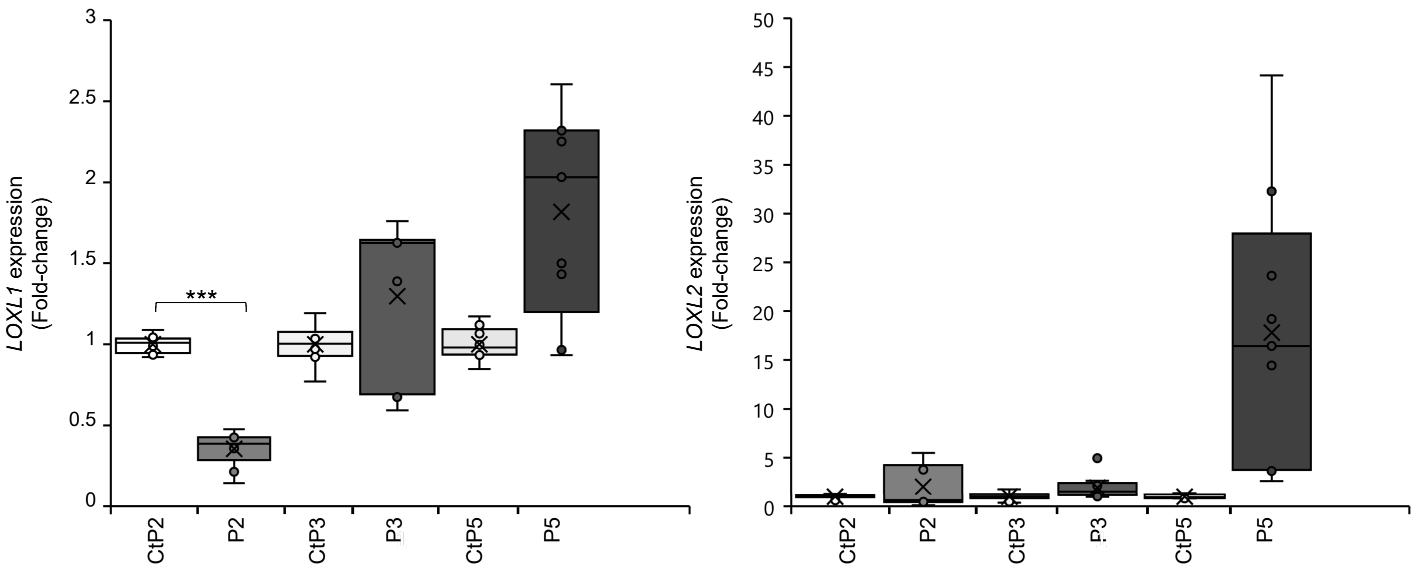

Figure 10. Analysis of gene expression in spEDS fibroblasts versus age and sex-matched controls. Results are presented as box plot with the mean symbolised by an X. Fold-changes were calculated for each patient using their corresponding control (n=3 experiments in triplicate); ***p<0.001 when comparing P2 to CtP2 (Student's t test).

Supplemental Figure 11

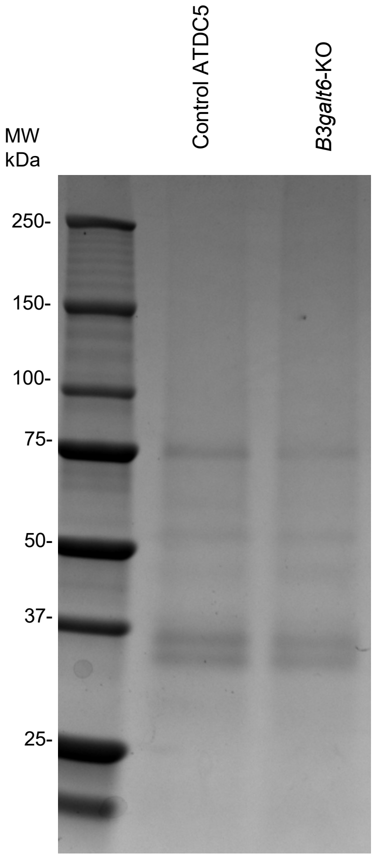

Figure 11. Comparison of collagen CNBr fragments obtained from control and *B3galt6*-KO ATDC5 pseudo-tissues. The collagen fraction was obtained by cell fractionation, digested with CNBr and analyzed by SDS-PAGE coupled to Coomassie staining. The image shown is representative of an analysis performed on 3 pseudotissues.

Supplemental Table 1

Table 1. Summary of patient characteristics and biochemical data from previous studies

Patient	DNA Change	Protein mutation	Syndrome subtype or disease	Residual in vitro β3GalT6 activity[1]	Residual GAG synthesis from 4-MUX[2]	HS expression[3]	Decorin glycanation[4]
**P2**	c.619G>C<br>c.619G>C	p.(D207H)<br>p.(D207H)	EDS-like	ND	60%	↘	↘↘↘
**P3**	c.619G>C<br>c.323_344del	p.(D207H)<br>p.(A108Gfs*163)	EDS-like	ND	16%	↘↘↘	↘↘↘
**P5**	c.649G>A<br>c.649G>A	p.(G217S)<br>p.(G217S)	EDS-like	ND	33%	↘	↘
**PVII.1**	c.545A>G<br>c.545A>G	p.(Y182C)<br>p.(Y182C)	SEMD-JL1	2%	19%	50%	↘↘↘

Data from (14) for patients P1, P2 and P3 and (16) for PVII.1

[1] Evaluated in vitro in patient fibroblast lysates using Gal-Xyl(2P)-OMN as acceptor substrate

[2] Measured in cellulo after Na$_2$[^{35}SO4]) radiolabeling in 4-MUX-primed GAG chains (10 µM)

[3] Visualised by immunofluorescence

[4] Evaluated by immunoblot from conditioned media from patient fibroblast cultures

ND stands for not determined and HS for heparan-sulfates.

Supplemental Table 2

Table 2. List of primers used for cloning β3GalT6-ΔN$_{ter}$29 or β3GalT6-ΔN$_{ter}$50 in the pETM41 expression vector and GlcAT-I-ΔN$_{ter}$76 in the pETM30 expression vector

**MBP-β3GalT6-ΔN$_{ter}$29-WT**	Sense	5′-CGCCATGGCACGCTGCGCGGCCGAGCCC-3′
	Antisense	5′-TGCTCGAGTCAGGGGATGCCCTCCCTTCTC-3′
**MBP-β3GalT6-ΔN$_{ter}$50-WT**	Sense	5′-CGCCATGGCAGCGCCCGCGCGCGCC-3′
	Antisense	5′-TGCTCGAGTCAGGGGATGCCCTCCCTTCTCTGGCA-3′
**GlcAT-I-ΔN$_{ter}$76-WT**	Sense	5′-TGCATAGCCATGGGAACG-3′
	Antisense	5′-TGCATGCGGCCGCTCA-3′
**MBP-ΔN$_{ter}$29-Y182C**	Sense	5′-CCGCCTCT**G**CTGGGGC-3′
	Antisense	5′-GCCCCAG**C**AGAGGCGG-3′
**MBP-ΔN$_{ter}$29-D207H**	Sense	5′-GGCAACTCGC**C**ACTACTACCT-3′
	Antisense	5′-AGGTAGTAGT**G**GCAGAGTTGCC-3′
**MBP-ΔN$_{ter}$29-G217S**	Sens	5′-GCGCTGGGCGGC**A**GCTACGTGCTCTC-3′
	Antisense	5′-GAGAGCACGTAGC**T**GCCGCCCAGCGC-3′

The three last sets of primers were used for site-directed mutagenesis using MBP-β3GalT6ΔN$_{ter}$29 construct. The mutated position is indicated in bold.
