# Supplementary material for: B3GALT6 mutations lead to compromised connective tissue biomechanics in Ehlers-Danlos syndrome
Source: JCI Insight. 2025 Aug 22;10(16):e179474. doi: 10.1172/jci.insight.179474 (PMC12406734; doi:10.1172/jci.insight.179474)

Full unedited gels for Figure 1B

The lanes shown in the figures of the manuscript are those that are framed.

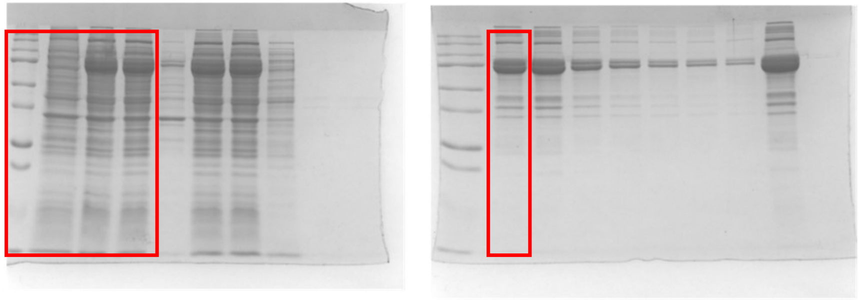

MBP-ΔN$_t$29-WT

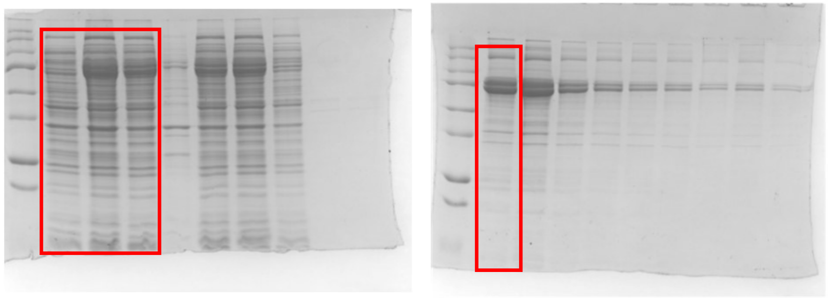

MBP-ΔN$_t$50-WT

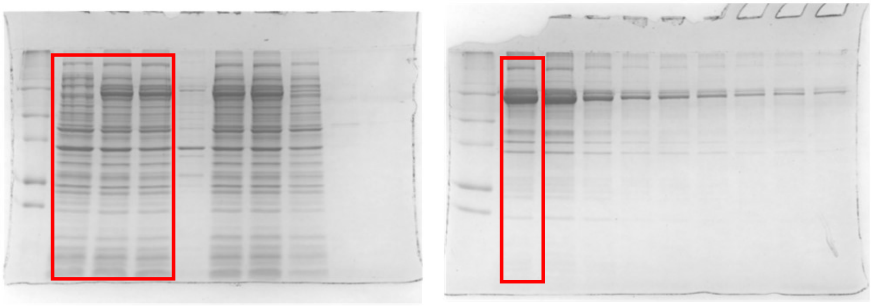

MBP-ΔN$_t$29-Y182C

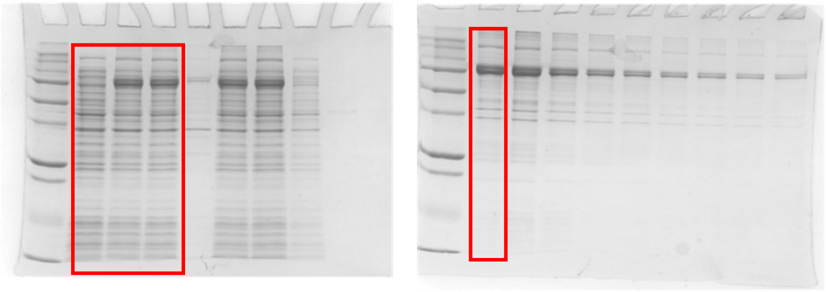

MBP-ΔN$_t$29-D207H

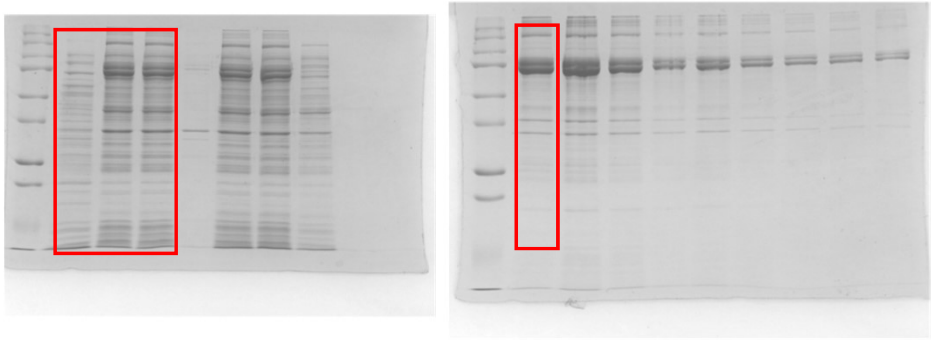

MBP-ΔN$_t$29-G217S

Full unedited gel for Figure 3E

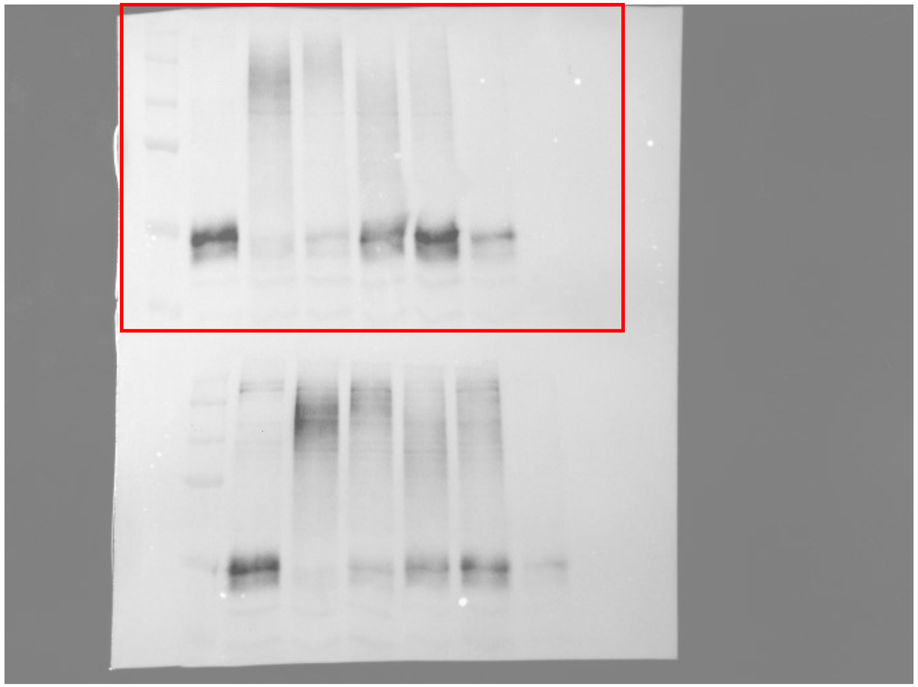

Full unedited gel for Figure 4A

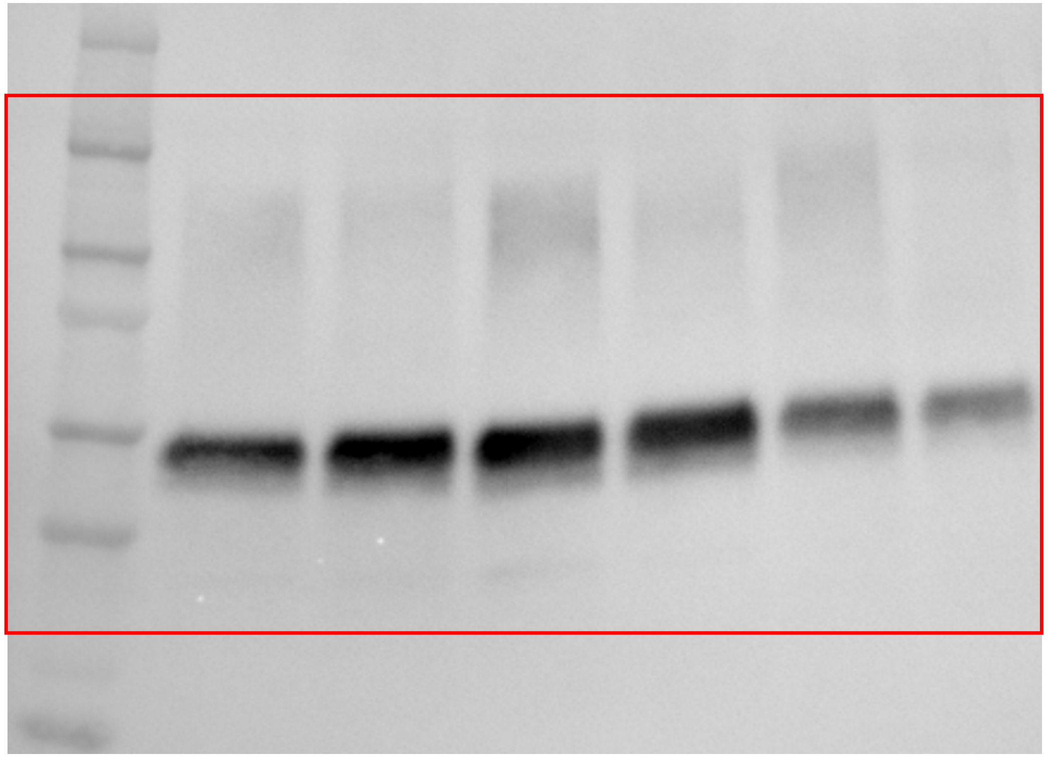

Full unedited gel for Figure 4D

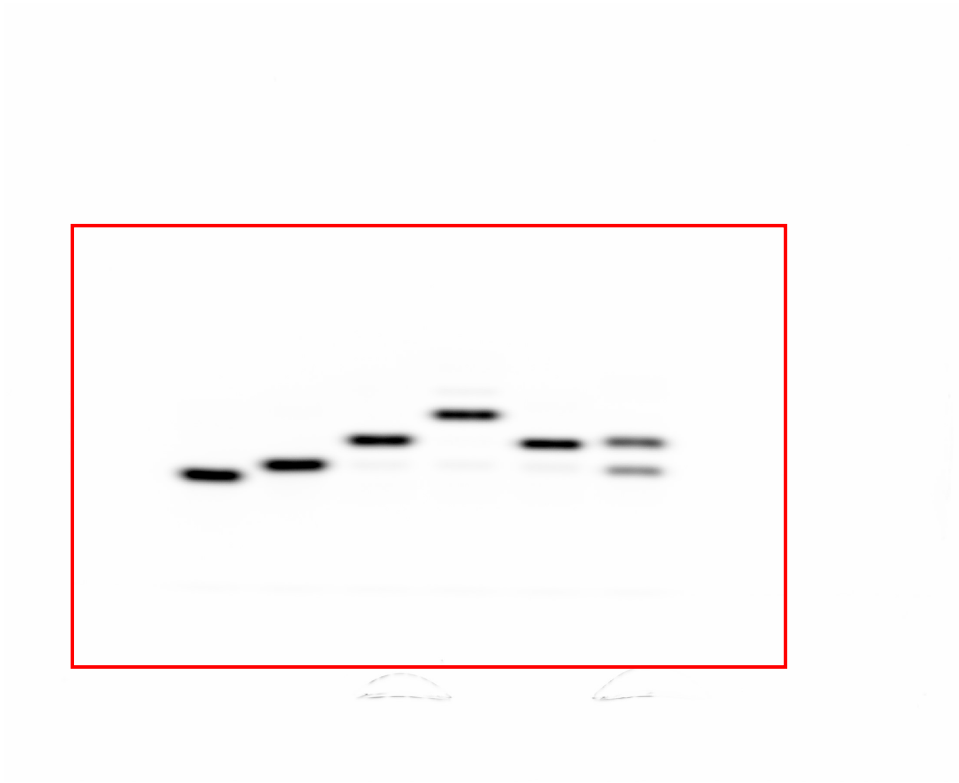

Full unedited gels for Figure 6A

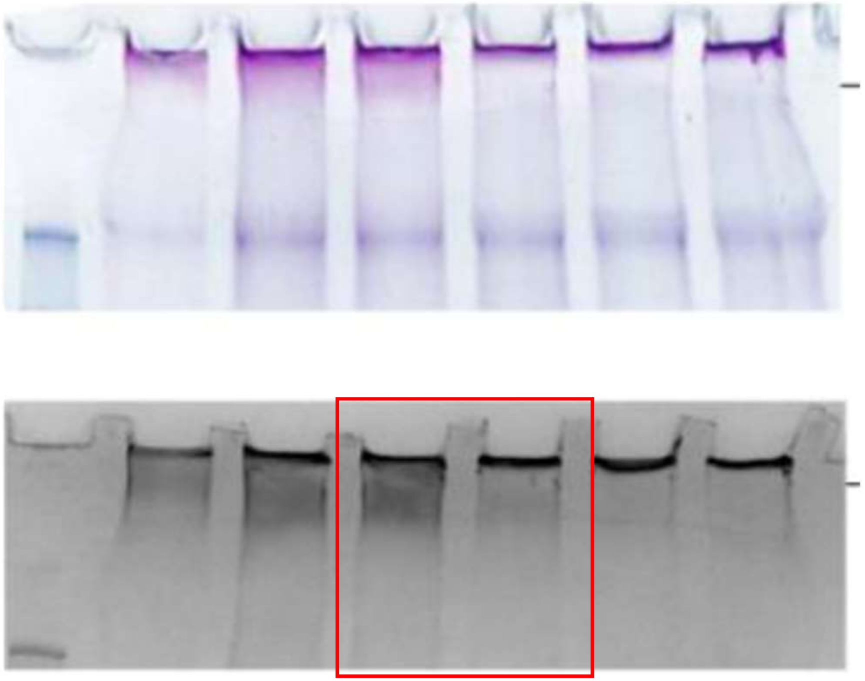

Full unedited gel for Figure 6B

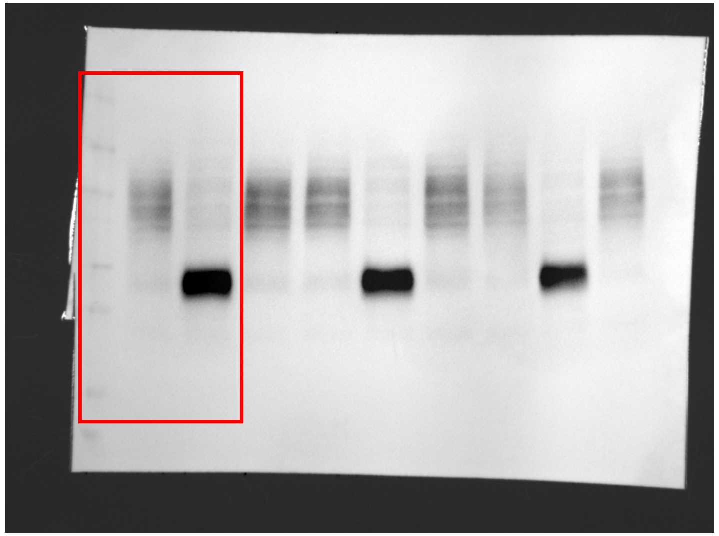

Full unedited gel for Figure 6C

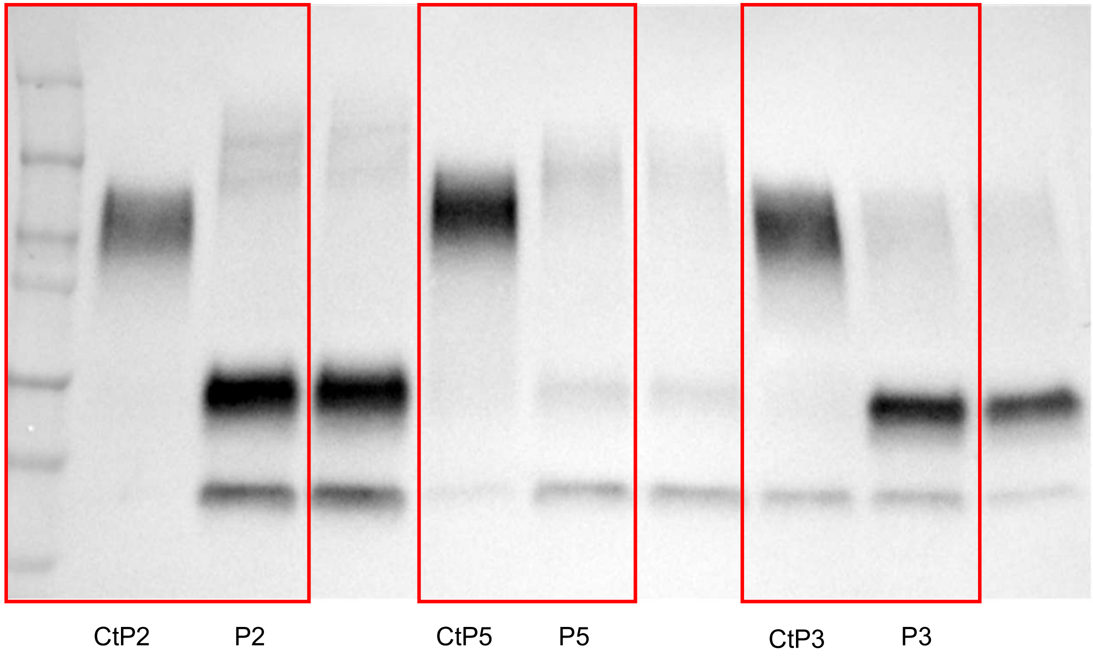

Full unedited gels for Figure 6D

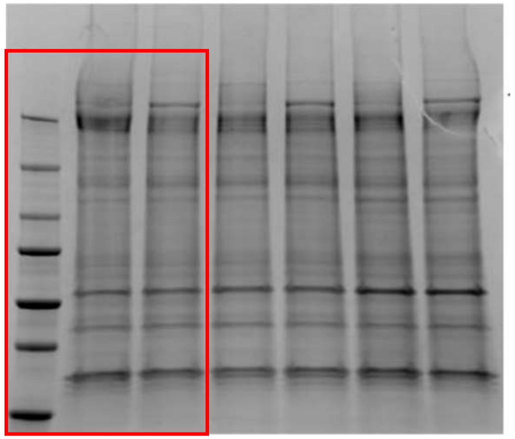

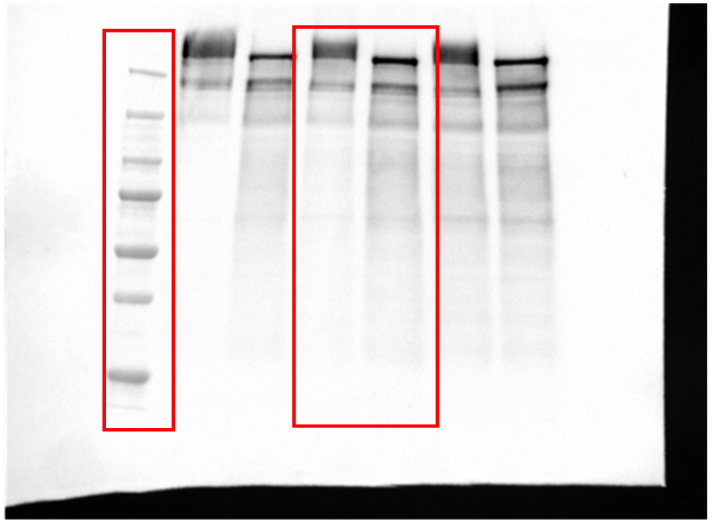

Full unedited gels for Supplemental Figure 8

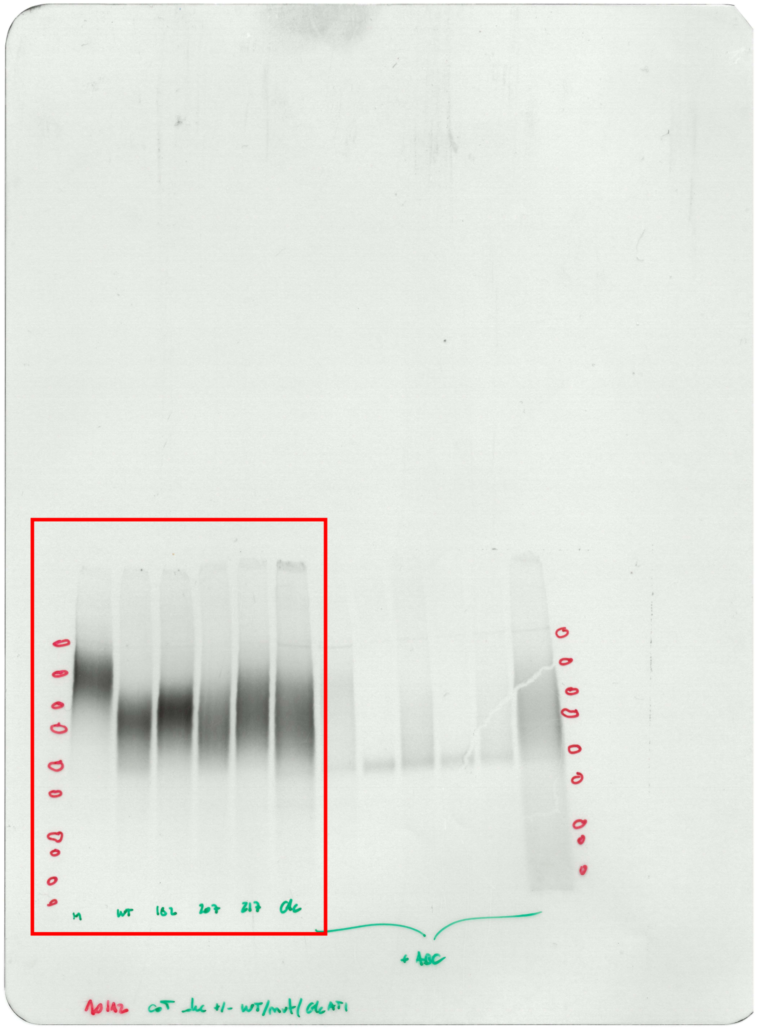

Full unedited gels for Supplemental Figure 11

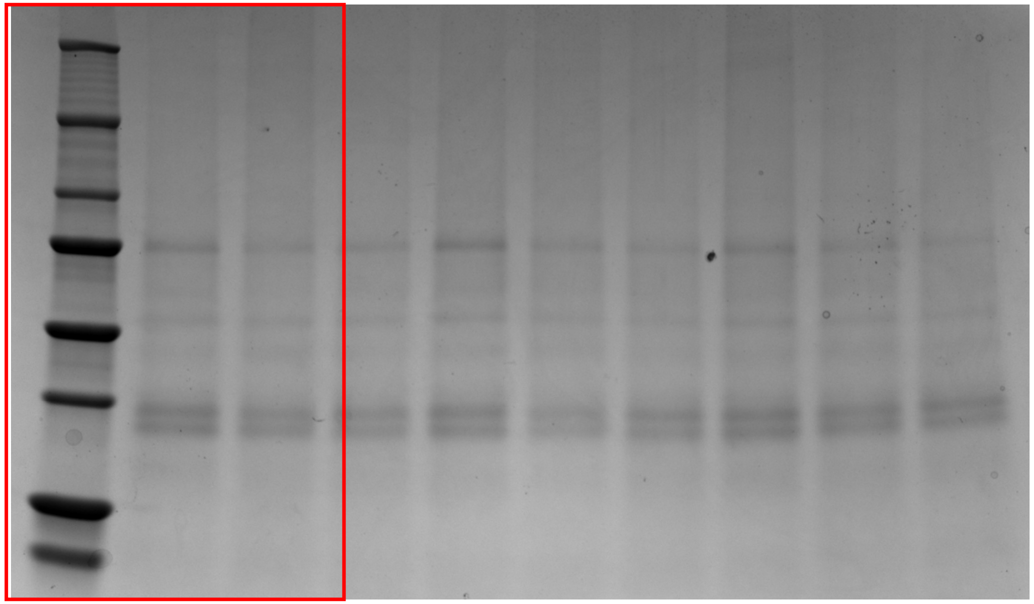

Supplement: Unedited blot and gel images [file jciinsight-10-179474-s280.pdf]
